# Prevalence and risk factors of ischemic stroke-related headache in China: a systematic review and meta-analysis

**DOI:** 10.1186/s12889-022-13917-z

**Published:** 2022-08-11

**Authors:** Qi Xie, Yinping Wu, Juhong Pei, Qianqian Gao, Qiang Guo, Xinglei Wang, Juanping Zhong, Yujie Su, Junqiang Zhao, Lanfang Zhang, Xinman Dou

**Affiliations:** 1 School of Nursing, Lanzhou University, Lanzhou, Gansu, China; 2 The Medical Department of Neurology, Lanzhou University Second Hospital, Lanzhou, Gansu, China; 3 The First Clinical Medical College, Lanzhou University, Lanzhou, Gansu, China; 4 Department of Nursing, Lanzhou University Second Hospital, Lanzhou, Gansu, China; 5 The Department of Tuberculosis Prevention and Control, Gansu Provincial Center for Disease Control and Prevention, Lanzhou, Gansu, China; 6 Department of Liver Diseases Branch, Lanzhou University Second Hospital, Lanzhou, Gansu, China; 7 School of Nursing University of Ottawa, Ottawa, Canada; 8 Department of Neurosurgery, Lanzhou University Second Hospital, Lanzhou, China

**Keywords:** Ischemic stroke, Headache, Prevalence, Risk factors, Systematic review, Meta-analysis

## Abstract

**Background:**

Headache accompanying ischemic stroke is considered an independent predictor of neurological deterioration. This meta-analysis aims to estimate the prevalence of ischemic stroke-related headaches and identify its risk factors in China.

**Methods:**

PubMed, Embase, Cochrane Library database, Web of Science, PsycINFO, and four Chinese databases for the related publications were searched. Two researchers independently selected the literature, extracted the relevant data, and assessed its methodological quality. The meta-analysis applied a random-effects model with R software to calculate the pooled prevalence of ischemic stroke-related headaches in Chinese patients, and to merge the odds ratio (OR) of risk factors. Subgroup analysis, sensitivity analysis, and meta-regression analysis were conducted. Publication bias was assessed by a funnel plot and Egger test.

**Results:**

Ninety-eight studies were eligible for inclusion. The overall pooled prevalence of ischemic stroke-related headache was 18.9%. Subgroup analysis showed that the prevalence of ischemic stroke related-headaches was higher among studies using self-report to diagnosis headache (18.9%; 95%CI, 8.9% to 40.2%), and those focused on age ≥ 55 years (19.7%; 95%CI, 14.9% to 25.9%), rural settings (24.9%; 95%CI, 19.7% to 31.6%). There were no significant differences in the headache prevalence between studies in the south and north, and inland and coastal studies. The prevalence of pre onset headache (13.9%) and tension-type headache (15.5%) and was higher compared with other types. History of headache (OR = 3.24; 95%CI, 2.26 to 4.65.), female gender (OR = 2.06; 95%CI, 1.44 to 2.96.), midbrain lesions (OR = 3.56; 95%CI, 1.86 to 6.83.), and posterior circulation stroke (OR = 2.13; 95%CI, 1.14 to 4.32) were major risk factors.

**Conclusion:**

The prevalence of ischemic stroke-associated headache is high in China. In addition, women, presence of midbrain lesions, posterior circulation stroke and a history of migraine were high-risk factors for ischemic stroke-related headaches. Designing effective interventions to prevent or alleviated headaches is necessary to promote patients’ neurological recovery and quality of life.

**Supplementary Information:**

The online version contains supplementary material available at 10.1186/s12889-022-13917-z.

## Background

Globally, stroke is the second leading cause of death [1] and poses a serious burden to the caregivers and society [2, 3]. Ischemic stroke accounts for more than 70% of strokes [4]. The focus of poststroke rehabilitation is usually on restoring neurological function and reducing the risk of recurrence. The presence of comorbidities, such as poststroke headache, is usually neglected and often undertreated, particularly in low- and middle-income countries [5]. Headache is a symptom of pain in the face, head, or neck, which can lead to disability in most patients with somatic and neurological disorders [6]. Headaches are usually divided into two types [7]: primary, which mainly include migraine and tension-type headaches (TTH) [8], and secondary, which are often caused by stroke, tumors, infections, etc. [9].

Headaches occur in 6%–44% of people with ischemic stroke [10]. Migraine with aura is associated with a two-fold increase in the risk for ischemic stroke [11, 12]. Additionally, headache accompanying ischemic stroke is considered an independent predictor of neurological deterioration [13, 14]. New-onset headache presenting with acute ischemic stroke is a predictor of persistent headache 6 months after stroke [15]. Poststroke headache is considered a common form of chronic poststroke pain [16, 17]. A previous systematic review has explored the global prevalence and characteristics of new-onset poststroke headache [10], within which only 2 of the 20 included studies were from Asian populations. However, in their review, neither did they perform a stratified analysis of the different types of headaches, nor a quantitative analysis of the additional risk factors was conducted, which limited our understanding of ischemic stroke-related headaches. Although the diverse study population in this review facilitated our understanding of the global status of ischemic stroke-related headaches, they failed to consider the national-level heterogeneities, within which the Chinese population has some unique features. According to the previous studies, China has the highest prevalence of stroke cases and bears the biggest stroke burden in the world [4, 18].

With demographic shifts and the rapid growth of China's elderly population, lifestyle habits in China are changing [19, 20]. Studies conducted in different regions of China have examined the prevalence of stroke-related headache symptoms. However, the reported prevalence varied widely from 0.6% [21] to 82.5% [22]. Moreover, the findings on the subgroups were inconsistent. For example, some studies have shown significant sex-specific differences in the prevalence of stroke-related headaches, in which women were found to be more prone to headaches than men [23, 24]. However, others have reported no such differences [25, 26]. Similarly, while some studies have shown that the prevalence of stroke-related headaches tends to decrease with age [27], others have reached an opposite conclusion [26, 28]. According to the data from the Global Burden of Disease Study, the incidence of stroke in China has decreased from 222/100,000 in 2005 to 201/100,000 in 2019 [29]. However, the prevalence of the disease continues to be on the rise [29].

Stroke-related headaches are more likely to be a significant cause of disability. The lack of epidemiological and outcome-based studies can limit the understanding and treatment of persistent poststroke headaches. Therefore, this study conducted this systematic review and meta-analysis to understand the prevalence and risk factors for stroke-related headaches in China, including Chinese and English language studies. In addition to estimating the overall prevalence of stroke-related headaches, we hypothesized that there would be differences in the prevalence of headaches based on differences in geographic setting, age, study setting, diagnostic methods, and headaches types. Furthermore, we conducted a meta-regression to explore the impact of the potential covariates such as methodological and economic factors on prevalence estimates. This work provides a strong theoretical basis for policy development on effective prevention and treatment services for this public health concern.

## Methods

This study was registered with PROSPERO (CRD42022328476) and conducted in accordance with the Preferred Reporting Items for Systematic Review and Meta-analysis (PRISMA) [30] guidelines.

### Search strategy

The following 9 electronic bibliographic databases were searched (from inception until December 30, 2021): PubMed, EMBASE, PsycINFO, Web of Science, Cochrane Library, CNKI, VIP, CBM, and the WanFang database for Chinese Periodicals, by applying a pretested search strategy.

Our search strategy employed medical subject heading (MeSH) and natural language text words. The references from the relevant papers or reviews were manually searched for additional studies. In case of missing relevant data from studies, we contacted the authors via email. Finally, all studies that were classified as headache studies among ischemic stroke patients in China were screened. On April 15, 2022, another search was performed on the previously mentioned database to locate the latest studies  (Supplementary Table 1).

### Inclusion and exclusion criteria

Studies were included in the review if they fulfilled the following inclusion criteria: observational studies (including cohort studies, cross-sectional studies, and case–control studies) that identified the prevalence of headaches in patients with ischemic stroke; studies that were published in English or Chinese language; studies that were published in a peer-reviewed journal or as conference proceedings with complete details. We excluded commentaries, letters, duplicate studies, reviews, and studies with a sample size below 60. Studies were also excluded if the full-text article was unable to be retrieved.

First, the Endnote X9 software was used to remove duplicates as well as to facilitate the screening process; second, the titles and abstracts in the non-duplicate papers were screened; and finally, the full texts were read to determine which studies were included/excluded, and the reasons for exclusion were recorded. The literature were independently screened by two researchers (Qi Xie and Qiang Guo) in accordance with the eligibility criteria. Any discrepancies were resolved through consensus or consultation with a third reviewer (Xin-Man Dou).

### Data extraction and quality assessment

The process of data extraction and quality assessment were conducted in duplicate (Qi Xie and Xinglei Wang) with third-party (Xin-Man Dou) adjudication for disagreements. Data from the included studies were extracted using a standard data extraction form. The following information was collected: first author, year of publication, geographical location (province and area), provincial Gross Domestic Product (GDP) (according to the Chinese government's administrative records), study setting (urban or rural), sample size, numbers of headache events, the characteristics of the study participants, types of headaches, and the diagnosis criteria of headache. If the number of headache events was not reported in the included studies, the proportion reported and the total sample size were used for analyses. To ascertain the risk factors for headache among patients with stroke in China, the odds ratio (OR) and associated 95% confidence intervals (CI) from multiple logistic regression were directly extracted from the included studies.

The methodological quality of case–control studies and cohort studies were assessed using the modified Newcastle–Ottawa Scale (NOS) [31]. The checklist consists of 5 items: representativeness of the sample, sample size, non-respondents, ascertainment of headache, and quality of descriptive statistics reporting. The total scores ranged from 0 to 5 points, with studies having a low risk of bias (≥ 3 points) or a high risk of bias (< 3 points) (Scoring details in supplementary Table 2). In addition, the risk of bias in a cross-sectional study was assessed using the instrument Agency for Healthcare Research and Quality (AHRQ) [32]. This tool had a total of 11 items, as listed below: if the answer to an object was “No” or “UNCLEAR,” the item’s score was “0”; if the answer was “Yes,” the item score “1”, with a total score of 0–11 points, 0–3 points = low quality, 4–7 points = medium quality, and 8–11 points = high quality [33].

### Statistical analyses

Meta-analysis was conducted using the meta () package available for the R software (version 4.1.2). Event rates and 95% CI were calculated for each study using the frequency of headaches reported in each study and the total sample size. To identify the risk factors for headache in Chinese ischemic stroke patients, the OR value was merged from the included studies. Based on the heterogeneity of the geographic regions and the variability in screening and diagnostic tools, we considered the random-effects model for meta-analysis as a better choice. A random-effects model was applied to assign weights to each study. Pooled effect sizes and event rates for each study were presented as a forest plot, where the size of each study was proportional to their weights. Statistical heterogeneity was quantified by the *I*^*2*^ statistic and formally tested by Cochran's Q statistic. Publication bias was assessed through visual inspection of a funnel plot and the result of the Egger test, considering statistically significant at *P* < 0.1. The robustness of the pooled estimates was assessed by sensitivity analysis (using leave-one-out analysis).

To explore the sources of heterogeneity, subgroup analyses were applied based on age (children < 18 years, adults 18–55 years, and elderly > 55 years), geographical setting (area), study setting (urban or rural), methods of diagnosis, and the types of headaches. Moreover, meta-regression analysis was performed to determine whether potential covariates could explain the heterogeneity between studies. Statistical significance was set at *P* < 0.05 [34]. To understand the impact of the China National Stroke Screening and Prevention Project (CNSSPP) [35] for high-risk stroke patients, which was released in 2012, the enrolled studies were divided into two categories based on their year of publication. This cut-off point was selected for studies before and after the year 2012. This cut-off point was selected based on the hypothesis that the implementation of the policy would affect the number of visits and the time to detection of the first clinical symptom [36].

## Results

### Study selection

**Fig. 1:**
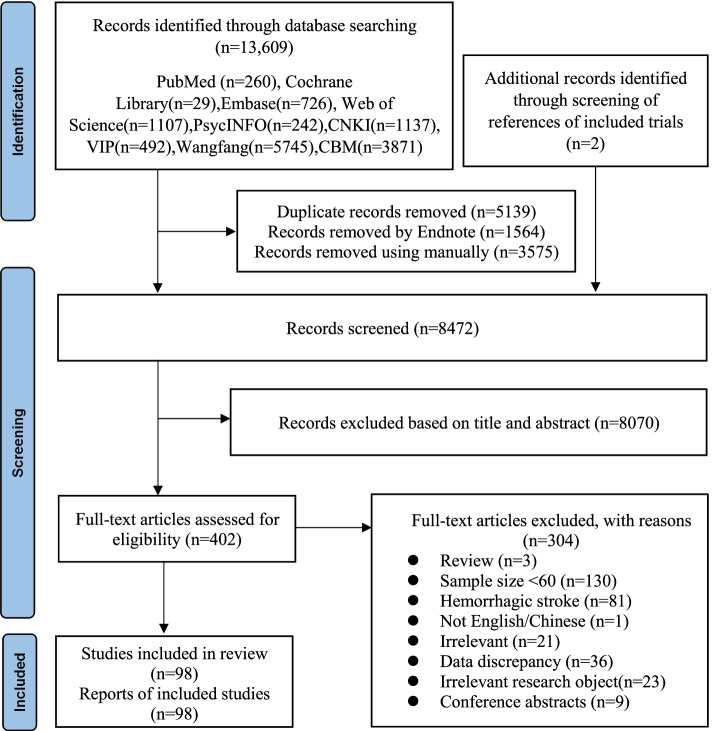
Flow diagram of the study selection process in the meta-analysis

**Fig. 2:**
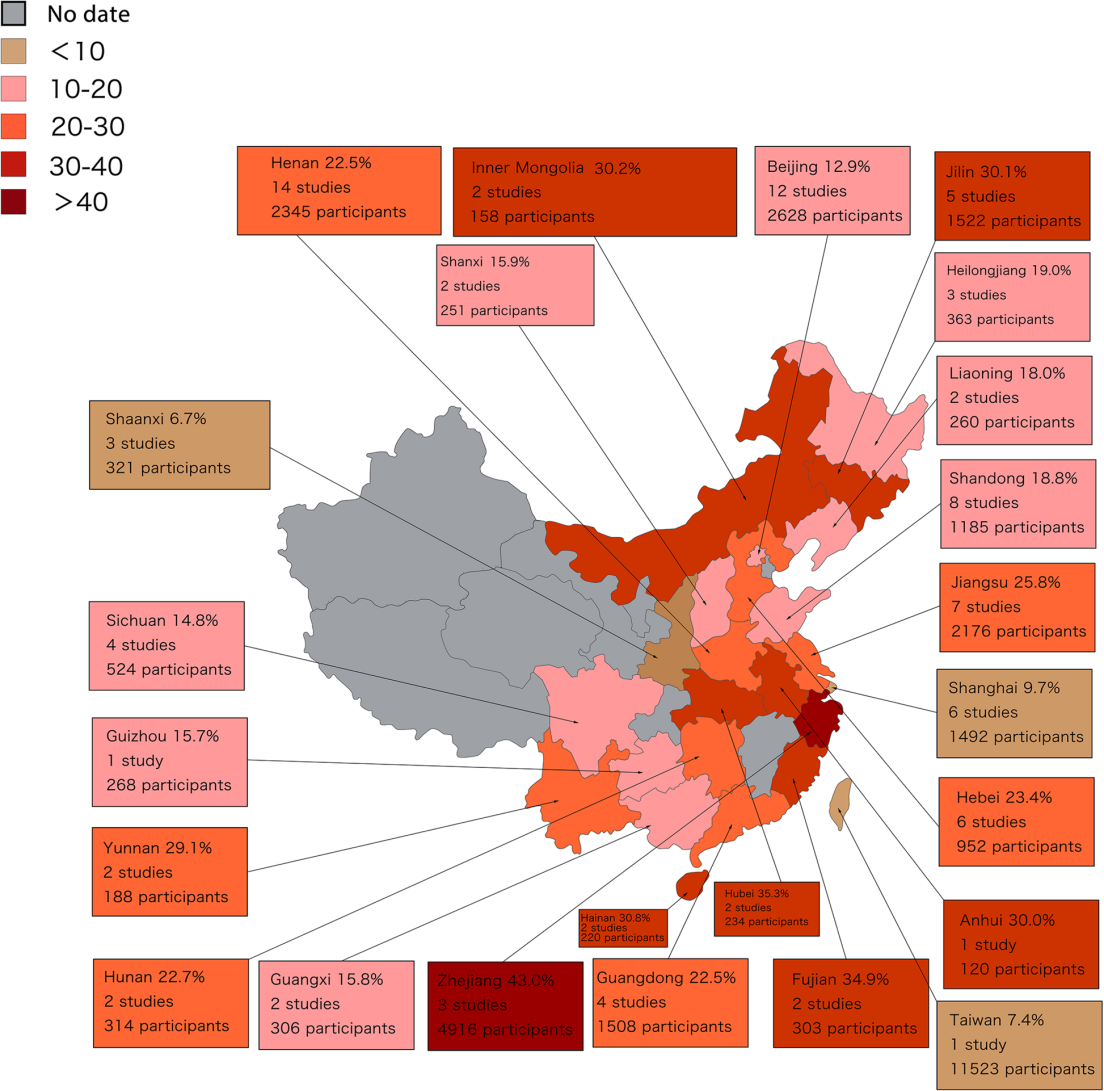
Provincial distribution pattern of ischemic stroke headache prevalence in China

In this study, 13,611 records were searched from the 9 databases and other resources (Fig. 1). After analyzing the title and abstract, 402 publications were selected for the full-text assessment. Finally, 98 full-text studies were included. A total of 98 studies from 24 regions in China were included in the meta-analysis, and the pooled sample size was 34,410 Chinese patients with ischemic stroke (Fig. 2).

### Study characteristics and methodologic quality

The 98 full-text studies that were included covered 22 provinces and 2 municipalities. Among the studies, 58 were conducted in northern China, 38 in the southern areas, and 2 studies did not specify the area. Furthermore, 74 studies were sourced from samples of the urban population, 18 studies from the rural population, 4 studies included both, and the remaining 2 studies did not mention the setting. Both coastal (*n* = 43) and inland areas (*n* = 53) were included. The method used for headache determination included a visual analog scale, self-reported, Guidelines for the Prevention and Treatment of Migraine in China, Migraine diagnostic criteria developed by the Collaborative Group on Epidemiological Investigation of Neurological Disorders, Select Committee of the National Institutes of Health, and the International Classification of Headache Disorders. For most of the studies, the source of the study population was single-center (*n* = 77, 78.6%) rather than multicenter (*n* = 6). According to the modified version of NOS scores and the AHRQ scores, 74 studies presented a relatively low risk of bias, whereas the remaining 24 presented a high risk of bias. (Supplementary Table 3).

### Meta-analysis of the pooled prevalence of headache

**Fig. 3:**
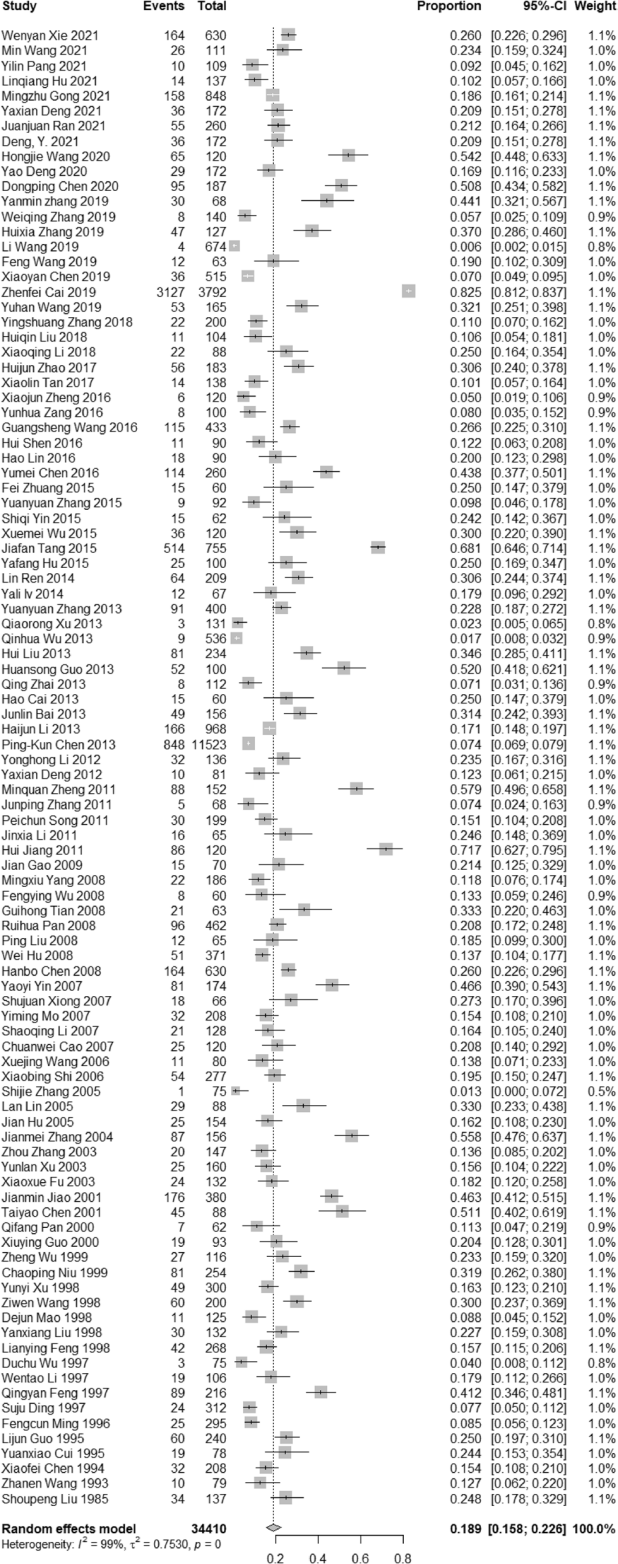
Forest plot of the prevalence of headaches

**Fig. 4:**
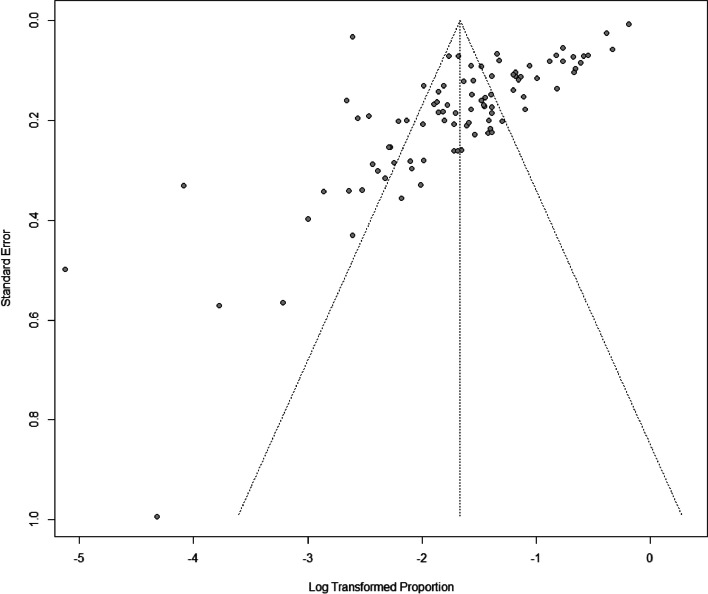
Funnel plot of the enrolled studies

The prevalence of headaches in the 98 studies varied widely from 0.6% to 82.5%. The pooled prevalence of headache among patients with ischemic stroke was 18.9% (95% *CI*: 15.8–22.6, *I*^2^ = 99%, Fig. 3). Table 1 summarized the subgroup pooled prevalence of headache among patients with ischemic stroke. The headaches were classified on the basis of headache types, location, duration, and site of cerebral infarction. The test for heterogeneity was significant in all the subgroups (*p* < 0.001) (Table 1). An obvious asymmetry in the funnel plot (Fig. 4) and Egger test (*p* < 0.1) showed the presence of significant publication bias. The results of the sensitivity analysis established that none of the studies had any significant impact on the pooled prevalence of headaches (Supplementary Fig. 1).

**Table 1:** Subgroup analyses of the prevalence of headache

Subgroup	No. of studies	No. of Total Participants	No. of Cases	Prevalence (%)	95%*CI*	Heterogeneity Test	
						*I*^*2*^* (%)*	*P value*
Type							
Migraine	18	5060	576	8.8	6.4–12.2	93	< 0.01
MA	3	1373	47	3.7	2.6–5.3	30	= 0.24
POH	16	6723	3508	13.9	8.0–23.9	99	< 0.01
IIH	6	12,978	944	7.2	5.4–9.7	79	< 0.01
PIH	7	1712	144	7.1	2.5–20.2	98	< 0.01
TTH	4	1018	156	15.5	8.5–28.1	94	< 0.01
Location							
One side	5	1533	101	5.8	2.1–15.9	96	< 0.01
Both sides	5	1361	86	6.2	3.3–11.5	88	< 0.01
Duration							
Intermittent pain	4	876	139	14.9	8.8–25.2	91	< 0.01
Persistent headache	3	689	60	7.8	3.7–16.7	88	< 0.01
Site of cerebral infarction							
Cortical infarction	6	1520	59	4.3	2.5–7.5	79	< 0.01
Vertebrobasilar artery	5	1483	101	5.3	2.5–11.2	92	< 0.01
Internal carotid artery	5	1483	153	8.8	3.5–22.1	97	< 0.01

*No* Number, *MA* Migraine with aura, *POH* Pre onset headache, *PIH* Post ictal headache, *IIH* Inter ictal headache, *TTH* Tension type headache

Subgroup analysis revealed that studies using self-report for diagnosis produced the highest prevalence of ischemic stroke headache (18.9%; *95% CI*, 8.9%–40.2%), followed by the visual analog scale (15.0%; *95% CI*, 3.4%–67.1%) and the International Classification of Headache Disorders (17.5%; *95% CI*, 7.7%–39.4%), and this difference was significant (*P* < 0.01). The prevalence of headaches did not differ between the southern and northern areas of China (*P* = 0.92); moreover, it did not differ between the inland and coastal regions (*P* = 0.94). The prevalence of headaches was the highest among patients with a mean age of ≥ 55 years (19.7%; *95% CI*, 14.9%–25.9%), followed by those ≤ 18 years of age (15.6%; *95% CI*, 11.8%–20.8%), and 18 to 55 years of age (13.9%; *95% CI*, 10.3%–18.8%). This difference was statistically significant (*P* = 0.02). Studies conducted in mixed settings reported the lowest prevalence of headache (10.0%; *95% CI*, 5.0%–20.1%) followed by urban settings (18.9%; *95% CI*, 15.9%–22.5%) and rural settings (24.9%; *95% CI*, 19.7%–31.6%). This subgroup difference was marginally statistically significant (*P* = 0.05).

Meta-regression analysis showed that the southern and northern areas (*P* = 0.70), inland and coastal regions (*P* = 0.53), provincial GDP (*P* = 0.39), and the year of publication (*P* = 0.59) were not significant sources of heterogeneity, whereas the quality assessment scores (*P* < 0.01) of studies and study setting (*P* = 0.04) were observed to be significant sources of heterogeneity.

### Risk factors for headache among patients with ischemic stroke

Three studies reported the risk factors associated with headache in ischemic stroke. Random-effects model analysis revealed that the risk for headache in patients with stroke who had a history of headache was 3.24 times higher than that in those without a history of headache. In the meta-analysis, the risk for headache in women with stroke was found to be 2.06 times higher than that in men. The prevalence of headache was 3.56-fold higher in strokes involving midbrain lesions, as reported by studies specifying the stroke location. Furthermore, the prevalence of headache was 2.13-fold higher in posterior circulation stroke, as reported by studies specifying the stroke location (Details in Table 2).

**Table 2:** Risk factors for headache in ischemic stroke patients in China

Variables	No. of studies	No. of Total Participants	No. of Cases	OR	95%*CI*	Heterogeneity Test	
						*I*^*2*^* (%)*	*P*
History of headache^a^	3	741	202	3.24	2.26–4.65	0	0.47
Female gender^a^	3	741	202	2.06	1.44–2.96	0	0.40
Midbrain lesions^a^	2	554	107	3.56	1.86–6.83	0	0.92
Posterior circulation stroke	1	11,523	848	2.13	1.14–4.32		

*No* Number, *a* analysis based on random-effects model

## Discussion

This meta-analysis was based on 34,410 subjects derived from 98 studies covering 24 provinces and municipalities in China, which enabled the reliable assessment of prevalence estimates of headaches at the national level. To the best of our knowledge, this is the first meta-analysis on the prevalence of headaches among patients with ischemic stroke in China, and the results demonstrated that the overall estimate of headache prevalence was 18.9%. This pooled prevalence is higher than that reported in previous studies for Asian and Middle Eastern (8%) [10] and North American (15%) [10] populations but lower than that reported for European populations (22%) [10]. Additionally, the prevalence is lower than that reported among patients with epilepsy (48%) [37] but higher than the reported prevalence of primary headache in a geriatric population (age > 60 years) in rural northern China (10.3%) [38]]. These variations in the headache prevalence could be attributed to the differences in the study population and the environment. Moreover, some comorbidities, such as common chronic diseases (e.g., diabetes), that cause vascular lesions and involve the corresponding nociceptive nerves may lead to an increased prevalence of headache in patients with ischemic stroke [39]. Combined with a decline in physical function with age, these factors may lead to a higher prevalence of ischemic stroke and headaches in people over the age of 55 [40]. This finding was also confirmed in our subgroup analysis on age, with the highest prevalence of headache being observed in people over 55 years of age. Additionally, most studies did not state whether standardized and validated measurement tools were used. Also, some patients were already comatose or aphasic and were unable to express their headache symptoms when they were sent to the emergency room [41]. Therefore, the prevalence of headache symptoms in patients with ischemic stroke may be higher than the results of the study. Therefore, we recommend early screening for ischemic stroke-related headaches in clinical practice.

Despite the availability of diagnostic criteria and classification tools for different headache types, the accurate diagnosis, and management of headache disorders remain challenging for nonexpert clinicians [42]. Therefore, a subgroup analysis was performed based on the headache screening tools to explore the prevalence of headaches in the different groups. Subgroup analysis showed that studies using self-report for diagnosis yielded the highest prevalence. However, self-reported diagnostic methods do not ensure the accurate classification and management of headaches [7]. Therefore, a tool that facilitates the diagnosis and management of chronic headache disorders by the clinicians involved in primary care needs to be developed.

Regarding the types of ischemic stroke-related headaches, migraine, pre onset headache (POH), and TTH were common types in the included studies, which was consistent with the results of a previous prospective study on headache at the onset of first ischemic stroke [43]. The pooled prevalences of migraine, POH, and TTH in patients with ischemic stroke were 8.8%, 13.9%, and 15.5%, respectively. The pooled prevalence of migraine was higher than the global prevalence of chronic migraine (0%–5.1%) in the general population [44]. This discrepancy could be because of the direct stimulation of the sensory afferents of the trigeminal vascular system by ischemic events or the indirect stimulation by ischemia-related factors [41, 45, 46]. Another possible cause is the ischemic infarction of the central pain conduction pathway [47]. Stratified analysis based on the region of cerebral infarction showed that the prevalence of headache was higher in the internal carotid artery system. However, the results should be cautiously interpreted, because only five studies have explored the relationship between headaches and the cerebral blood supply system. Therefore, more studies are required to explore the prevalence of headaches in the cerebral blood supply system and to confirm whether regional differences exist.

The findings from our research indicated that the study setting may influence the incidence of headaches. Patients with ischemic stroke who hailed from urban areas appeared to face less risk for headaches than those from rural areas. First, the distribution of stroke disease burden in China exhibited significant urban–rural differences [36]. The National Health Service Survey data for the period 1993–2013 showed that the prevalence of stroke in rural areas was significantly lower than that in urban areas. However, since 2013, the prevalence of stroke in rural areas has increased rapidly and has surpassed that in urban areas, and the difference was more significant in 2018 [48]. From 2010 to 2019, there was no significant change in the overall crude mortality rate of stroke in urban areas, whereas that in rural areas demonstrated an increasing trend and was much higher than that of urban residents during the same period [36]. All these factors are more likely to increase the risk of headaches in patients from rural areas. Second, significant national differences existed in the accessibility and quality of stroke care [5] Relative to rural areas, patients from urban areas enjoyed relatively greater access to care that met key organizational and staffing parameters (e.g., separate wards, staff dedicated to stroke care, regular multidisciplinary team meetings, established care protocols, staff education and training, and educational information for patients and caregivers) [49–51]. This finding highlights the importance of stroke management in rural areas. In the future, the Chinese government should increase the number of organizations that fulfill the accepted standards of care for global outcomes and conduct early screening in rural as soon as possible.

In China, the burden of stroke is geographically distributed as “high in the north and low in the south” and the mortality-to-incidence ratio is the lowest (suggesting a greater abundance of relevant medical resources) in economically developed regions, such as the eastern and southern coasts [36]. However, significant differences were not observed in this study in the prevalence of headaches based on subgroup analysis in the southern and northern regions as well as coastal and inland regions. This difference may be due to the influence of other factors, such as the patient's original body condition, the site of the ischemic stroke lesion, and the associated pathophysiological mechanisms of headache [41, 45–47], which are more important than the regional factors in the occurrence of headache in patients. Therefore, future studies should attempt to identify the greatest risk factors for a headache that are linked to patients with ischemic stroke in China and, thus, provide theoretical guidance for effective prevention and interventions.

Finally, our study revealed that women were independent predictors of the occurrence of ischemic stroke-related headaches, which is consistent with the results of a previous study on migrainous infarction [52]. Primarily, this finding may be related to the endocrine hormones and physiological protein regulation in women [53]. Second, women are more susceptible to mood swings than men and are especially more likely to experience negative emotions, such as anxiety and irritability, because of an illness. All these factors may exacerbate the risk for headaches in women. Furthermore, the results of this study demonstrated that a history of migraine was an independent risk factor for the development of ischemic stroke-related headaches. It is currently accepted that biochemical alterations, such as the aggregation of excitatory amino acids (glutamate and aspartate), are involved in the excitation of the migraine center in the mechanism of migraine [54] and that these biochemical alterations occur in ischemic stroke [55]. Additionally, during the acute phase of ischemic stroke, the pathophysiological process of vasoconstriction is caused by the release of inflammatory transmitters, such as cytokines and vasoactive peptides, the upregulation of adhesion molecules, and the release of potassium from depolarized nerve cells occur during migraine attacks [55]. Therefore, ischemic stroke-associated migraine may be related to pre-existing migraine being triggered. Another important finding is that the midbrain was an independent predictor of headache onset, which is consistent with the results of a previous prospective study on lacunar cerebral infarction [39]. As the pathophysiological basis of the conduction pathways and the mechanisms in the central pain continue to be elucidated, it can be hypothesized that central pain may be related to the damage of the midbrain periaqueductal gray. This important structure is involved in pain conduction and regulation and may play a key role in headache onset. However, this finding needs to be confirmed with further studies involving larger sample sizes and by combining imaging, electrophysiological, and pathophysiological methods. Similarly, posterior circulation stroke was found to be a predictor of headache onset, which is consistent with the results of a previous prospective study on lacunar cerebral infarction [43]. Although posterior circulation stroke is a risk factor, the small number of studies did not allow for meta-analysis; therefore, more prospective original studies are needed to validate the findings.

### Strength and limitations

Our review is the first meta-analysis on the prevalence of ischemic stroke-related headaches in China. The study has the following strengths: (1) the review was conducted on a large number of participants, ensuring the statistical power and accuracy of the estimates; (2) numerous studies included in the meta-analysis were described in Chinese, hence pooling of these data may be considered valuable to non-Chinese readers and for future studies on ischemic stroke-related headache and the related fields; (3) despite differences in in the demographic characteristics and methods, such as the diagnostic criteria for headache, the sensitivity analysis suggested that our final pooled results are statistically robust. Nevertheless, our study has the following limitations. The potential sources of heterogeneity were explored through subgroup analysis and meta-regression analysis. However, considerable heterogeneity remained in the studies evaluated in the subgroup analysis, as it is usually difficult to avoid heterogeneity in epidemiological studies [56]. In addition, despite our efforts to avoid publication bias (i.e., searching both English and Chinese databases for articles, including peer-reviewed articles), publication bias occurred, which needs to be considered when interpreting the study outcomes.

## Conclusions

The results from the present study establish that the prevalence of ischemic stroke-associated headaches is high in China. Compared with migraine, migraine with aura (MA), inter ictal headache (IIH) and post ictal headache (PIH), the pooled prevalence of POH and TTH was higher. The prevalence of ischemic stroke related-headaches varied significantly according to the different diagnosis criteria, age, and study settings. However, there were no significant differences in the headache prevalence between studies in the south and north, and inland and coastal studies. Additionally, women, those with midbrain lesions, those with posterior circulation stroke, and patients with a history of migraine were at a higher risk for ischemic stroke-related headaches. The prevalence of stroke is high in China, the country has a large rural population, and headache is associated with the functional recovery of the nervous system. Considering these factors, there is an urgent need for policymakers and healthcare providers at the national and regional levels to implement early screening programs and develop effective prevention and intervention measures.

## Supplementary Information

**Additional file 1.**

**Additional file 2.**

**Additional file 3.**

**Additional file 4.**

## Data Availability

All data generated or analysed during this study are included in this published article [and its supplementary information files].

## Abbreviations

TTH: Tension-type headaches
PRISMA: The Preferred Reporting Items for Systematic Review and Meta-analysis
CNKI: The China National Knowledge Infrastructure
VIP: The VIP Database for Chinese Technical Periodicals
CBM: The China Biomedical Literature Database
MeSH: Medical subject heading
GDP: Provincial Gross Domestic Product
OR: The odds ratio
CI: Confidence intervals
NOS: Newcastle–Ottawa Scale
AHRQ: The instrument Agency for Healthcare Research and Quality
CNSSPP: The China National Stroke Screening and Prevention Project
MA: Migraine with aura
POH: Pre onset headache
PIH: Post ictal headache
IIH: Inter ictal headache
