# Supplementary figures and images for: Prevalence and risk factors of ischemic stroke-related headache in China: a systematic review and meta-analysis
Source: BMC Public Health. 2022 Aug 11;22:1533. doi: 10.1186/s12889-022-13917-z (PMC9367127; doi:10.1186/s12889-022-13917-z)

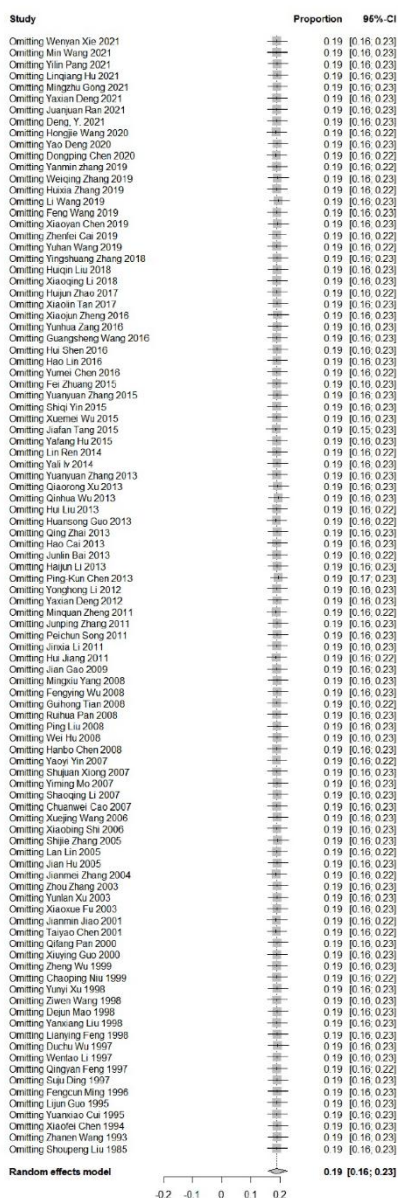

Supplementary figure 1. Sensitivity analysis of prevalence of headache among patients

Supplement: Supplementary file 1 — Additional file 1. [file 12889_2022_13917_MOESM1_ESM.pdf]
