# Supplementary material for: Prevalence and risk factors of ischemic stroke-related headache in China: a systematic review and meta-analysis
Source: BMC Public Health. 2022 Aug 11;22:1533. doi: 10.1186/s12889-022-13917-z (PMC9367127; doi:10.1186/s12889-022-13917-z)
Supplement: Supplementary file 2 — Additional file 2. [file 12889_2022_13917_MOESM2_ESM.pdf]

Supplementary table 1 search strategy

PubMed: from inception to April 15, 2022

#	searches	results
1	"Stroke"[MeSH Terms] OR "Carotid Artery Diseases"[MeSH Terms] OR "Cerebrovascular Disorders"[MeSH Terms] OR "Basal Ganglia Cerebrovascular Disease"[MeSH Terms] OR "Cerebral Infarction"[MeSH Terms] OR "Brain Ischemia"[MeSH Terms] OR "Cerebral Small Vessel Diseases"[MeSH Terms] OR "Intracranial Arterial Diseases"[MeSH Terms] OR "Intracranial Hemorrhages"[MeSH Terms] OR "Brain Infarction"[MeSH Terms] OR "stroke, lacunar"[MeSH Terms] OR "vasospasm, intracranial"[MeSH Terms] OR "Hemiplegia"[MeSH Terms] OR "Paresis"[MeSH Terms] OR "gait disorders, neurologic"[MeSH Terms]	424,554
2	stroke*[Title/Abstract] OR "Cerebrovascular Accident*"[Title/Abstract] OR "Cerebrovascular Apoplexy"[Title/Abstract] OR "Brain Vascular Accident*"[Title/Abstract] OR "Cerebrovascular Stroke*"[Title/Abstract] OR "Apoplexy"[Title/Abstract] OR "Cerebral Stroke*"[Title/Abstract] OR "Acute Stroke*"[Title/Abstract] OR "Acute Cerebrovascular Accident*"[Title/Abstract] OR "Brain Stem Infarctions"[Title/Abstract] OR "Cerebral Infarction"[Title/Abstract] OR "Hemorrhagic Stroke"[Title/Abstract] OR "Ischemic Stroke"[Title/Abstract] OR "Embolic Stroke"[Title/Abstract] OR "Thrombotic Stroke"[Title/Abstract] OR "Cardiovascular Diseases"[Title/Abstract] OR "Vascular Diseases"[Title/Abstract] OR "Carotid Artery Disease*"[Title/Abstract] OR "Carotid Artery	500,380

	Disorder*"[Title/Abstract] OR "Carotid Arterial Disease*"[Title/Abstract] OR"Carotid Atheroscleros*"[Title/Abstract] OR "Carotid Atherosclerotic Disease*"[Title/Abstract] OR "Internal Carotid Artery Diseases"[Title/Abstract] OR Common Carotid Artery Diseases[Title/Abstract] OR External Carotid Artery Diseases[Title/Abstract] OR External Carotid Arterial Diseases[Title/Abstract] OR "Carotid Artery Thrombosis"[Title/Abstract] OR "Cerebrovascular Disorder*"[Title/Abstract] OR "Intracranial Vascular Disease*"[Title/Abstract] OR "Intracranial Vascular Disorder*"[Title/Abstract] OR "Cerebrovascular Disease*"[Title/Abstract] OR "Brain Vascular Disorder*"[Title/Abstract] OR "Cerebrovascular Occlusion*"[Title/Abstract] OR "Cerebrovascular Insufficienc*"[Title/Abstract] OR "Basal Ganglia Vascular Disease*"[Title/Abstract] OR "Lenticulostriate Vasculopath*"[Title/Abstract] OR Lenticulostriate Vascular Disease*[Title/Abstract] OR Vascular Lenticulostriate Diseases[Title/Abstract] OR "Basal Ganglia Hemorrhage"[Title/Abstract] OR "Putaminal Hemorrhage"[Title/Abstract] OR "Cerebral Infarct*"[Title/Abstract] OR "Left Hemisphere Cerebral Infarction"[Title/Abstract] OR "Subcortical Infarction*"[Title/Abstract] OR "Posterior Choroidal Artery Infarction"[Title/Abstract] OR "Anterior Choroidal Artery Infarction"[Title/Abstract] OR "Right Hemisphere Cerebral Infarction"[Title/Abstract] OR "CADASIL"[Title/Abstract] OR "Multi-Infarct Dementia"[Title/Abstract] OR "Anterior Cerebral	

	Artery Infarction"[Title/Abstract] OR "Middle Cerebral Artery Infarction"[Title/Abstract] OR "Posterior Cerebral Artery Infarction"[Title/Abstract] OR "Brain Ischemia*"[Title/Abstract] OR "Ischemic Encephalopath*"[Title/Abstract] OR "Cerebral Ischemia*"[Title/Abstract] OR "Brain Hypoxia-Ischemia"[Title/Abstract] OR "Cerebral Small Vessel Disease*"[Title/Abstract] OR "Cerebral Microangiopath*"[Title/Abstract] OR "Intracranial Arterial Disease*"[Title/Abstract] OR "Intracranial Arterial Disorder*"[Title/Abstract] OR "Arterial Brain Disease*"[Title/Abstract] OR Brain Arterial Disease*[Title/Abstract] OR Arterial Brain Disorder*[Title/Abstract] OR "Intracranial Hemorrhage*"[Title/Abstract] OR "Posterior Fossa Hemorrhage*"[Title/Abstract] OR "Brain Hemorrhage*"[Title/Abstract] OR "Cerebral Hemorrhage"[Title/Abstract] OR "Cerebral Intraventricular Hemorrhage"[Title/Abstract] OR "Hypertensive Intracranial Hemorrhage"[Title/Abstract] OR "Cranial Epidural Hematoma"[Title/Abstract] OR "Subdural Hematoma"[Title/Abstract] OR "Pituitary Apoplexy"[Title/Abstract] OR "Subarachnoid Hemorrhage"[Title/Abstract] OR "Brain Infarction*"[Title/Abstract] OR "Brain Infarct*"[Title/Abstract] OR "Anterior Circulation Brain Infarction"[Title/Abstract] OR "Brain Venous Infarction*"[Title/Abstract] OR "Anterior Cerebral Circulation Infarction"[Title/Abstract] OR "Posterior Circulation Brain Infarction"[Title/Abstract] OR "Lacunar Stroke*"[Title/Abstract] OR "Lacunar Syndrome*"[Title/Abstract]	

	OR "Lacunar Infarction*"[Title/Abstract] OR "Lacunar Infarct*"[Title/Abstract] OR "Intracranial Vasospasm*"[Title/Abstract] OR Intracranial Angiospasm*[Title/Abstract] OR "Intracranial Vascular Spasm*"[Title/Abstract] OR "Cerebral Vasospasm*"[Title/Abstract] OR "Cerebrovascular Spasm*"[Title/Abstract] OR "Cerebral Angiospasm*"[Title/Abstract] OR "Cerebral Artery Spasm*"[Title/Abstract] OR "Hemiplegia*"[Title/Abstract] OR "Transient Hemiplegia*"[Title/Abstract] OR "Monoplegia*"[Title/Abstract] OR "Post-Ictal Hemiplegia*"[Title/Abstract] OR "Crossed Hemiplegia*"[Title/Abstract] OR "Flaccid Hemiplegia*"[Title/Abstract] OR "Infantile Hemiplegia*"[Title/Abstract] OR "Spastic Hemiplegia*"[Title/Abstract] OR "Pareses"[Title/Abstract] OR "Paraparesis"[Title/Abstract] OR "Muscular Pares*"[Title/Abstract] OR "Muscle Pares*"[Title/Abstract] OR "Monopares*"[Title/Abstract] OR "Lower Extremity Pares*"[Title/Abstract] OR "Crural Pares*"[Title/Abstract] OR "Upper Extremity Pares*"[Title/Abstract] OR "Brachial Pares*"[Title/Abstract] OR "Hemipares*"[Title/Abstract] OR "Spastic Paraparesis"[Title/Abstract] OR "Neurologic Gait Disorder*"[Title/Abstract] OR Neurologic Locomotion Disorder*[Title/Abstract] OR Neurologic Ambulation Disorder*[Title/Abstract] OR "Neurologic Gait Dysfunction*"[Title/Abstract] OR "Duck Gait"[Title/Abstract] OR Sensorimotor Gait Disorder*[Title/Abstract] OR Athetotic Gait[Title/Abstract] OR Broadened Gait[Title/Abstract] OR "Drop	

	Foot Gait"[Title/Abstract] OR "Festinating Gait"[Title/Abstract] OR "Frontal Gait"[Title/Abstract] OR "Hemiplegic Gait"[Title/Abstract] OR "Hysterical Gait"[Title/Abstract] OR Reeling Gait[Title/Abstract] OR "Rigid Gait"[Title/Abstract] OR "Scissors Gait"[Title/Abstract] OR "Shuffling Gait*"[Title/Abstract] OR "Spastic Gait"[Title/Abstract] OR "Stumbling Gait"[Title/Abstract] OR "Unsteady Gait"[Title/Abstract] OR Widebased Gait[Title/Abstract] OR "Marche a Petit Pas"[Title/Abstract] OR Rapid Fatigue Gait[Title/Abstract] OR Charcot Gait*[Title/Abstract] OR Charcot* Gait[Title/Abstract] OR "Gait Apraxia"[Title/Abstract] OR "Gait Ataxia"[Title/Abstract]	
3	#1 OR #2	685,918
4	"Headache"[Mesh]	29,884
5	Headache*[Title/Abstract] OR "Head Pain*"[Title/Abstract] OR Cephalodynia*[Title/Abstract] OR "Cranial Pain*"[Title/Abstract] OR Cephalalgia*[Title/Abstract] OR Cephalgia*[Title/Abstract] OR "Generalized Headache*"[Title/Abstract] OR "Ocular Headache*"[Title/Abstract] OR "Orthostatic Headache*"[Title/Abstract] OR "Vertex Headache*"[Title/Abstract] OR "Retro-Ocular Headache*"[Title/Abstract] OR "Retro Ocular Headache*"[Title/Abstract] OR "Sharp Headache*"[Title/Abstract] OR "Throbbing Headache*"[Title/Abstract] OR "Unilateral Headache*"[Title/Abstract] OR Hemicrania[Title/Abstract] OR "Bilateral Headache*"[Title/Abstract] OR "Periorbital Headache*"[Title/Abstract] OR "Slit Ventricle Syndrome"[Title/Abstract]	96,435
6	#4 OR #5	104,703

7	China[Title/Abstract] OR Chinese[Title/Abstract] OR Guangxi[Title/Abstract] OR "inner Mongolia"[Title/Abstract] OR Ningxia[Title/Abstract] OR Tibet[Title/Abstract] OR Xizang[Title/Abstract] OR Xinjiang[Title/Abstract] OR Heilongjiang[Title/Abstract] OR Jilin[Title/Abstract] OR Liaoning[Title/Abstract] OR Hebei[Title/Abstract] OR Shanxi[Title/Abstract] OR Shandong[Title/Abstract] OR Shaanxi[Title/Abstract] OR Gansu[Title/Abstract] OR Qinghai[Title/Abstract] OR Sichuan[Title/Abstract] OR Hubei[Title/Abstract] OR Hunan[Title/Abstract] OR Henan[Title/Abstract] OR Anhui[Title/Abstract] OR Zhejiang[Title/Abstract] OR Jiangsu[Title/Abstract] OR Guangdong[Title/Abstract] OR Jiangxi[Title/Abstract] OR Fujiang[Title/Abstract] OR Guizhou[Title/Abstract] OR Yunnan[Title/Abstract] OR Hainan[Title/Abstract] OR Han[Title/Abstract] OR Hui[Title/Abstract] OR Mongolia[Title/Abstract] OR Zhuang[Title/Abstract] OR Taiwan[Title/Abstract] OR "Hong Kong"[Title/Abstract] OR Macau[Title/Abstract]	517,659
8	#3 AND #6 AND #7	260

Cochrane database Library：from inception to April 15, 2022

#	searches	results
1	[mh "Stroke"] OR [mh "Carotid Artery Diseases"] OR [mh "Cerebrovascular Disorders"] OR [mh "Basal Ganglia Cerebrovascular Disease"] OR [mh "Cerebral Infarction"] OR [mh "Brain Ischemia"] OR [mh "Cerebral Small Vessel Diseases"] OR [mh "Intracranial Arterial Diseases"] OR [mh "Intracranial Hemorrhages"] OR [mh "Brain Infarction"] OR [mh "stroke, lacunar"] OR [mh "vasospasm, intracranial"] OR [mh	18114

	"Hemiplegia"] OR [mh "Paresis"] OR [mh "gait disorders, neurologic"]	
2	(stroke* OR Cerebrovascular Accident* OR Cerebrovascular Apoplexy OR Brain Vascular Accident* OR Cerebrovascular Stroke* OR Apoplexy OR Cerebral Stroke* OR Acute Stroke* OR Acute Cerebrovascular Accident* OR Brain Stem Infarctions OR Cerebral Infarction OR Hemorrhagic Stroke OR Ischemic Stroke OR Embolic Stroke OR Thrombotic Stroke OR Cardiovascular Diseases OR Vascular Diseases OR Carotid Artery Disease* OR Carotid Artery Disorder* OR Carotid Arterial Disease* OR Carotid Atheroscleros* OR Carotid Atherosclerotic Disease* OR Internal Carotid Artery Diseases OR Common Carotid Artery Diseases OR External Carotid Artery Diseases OR External Carotid Arterial Diseases OR Carotid Artery Thrombosis OR Cerebrovascular Disorder* OR Intracranial Vascular Disease* OR Intracranial Vascular Disorder* OR Cerebrovascular Disease* OR Brain Vascular Disorder* OR Cerebrovascular Occlusion* OR Cerebrovascular Insufficienc* OR Basal Ganglia Vascular Disease* OR Lenticulostriate Vasculopath* OR Lenticulostriate Vascular Disease* OR Vascular Lenticulostriate Diseases OR Basal Ganglia Hemorrhage OR Putaminal Hemorrhage OR Cerebral Infarct* OR Left Hemisphere Cerebral Infarction OR Subcortical Infarction* OR Posterior Choroidal Artery Infarction OR Anterior Choroidal Artery Infarction OR Right Hemisphere Cerebral Infarction OR CADASIL OR Multi-Infarct Dementia OR Anterior Cerebral Artery Infarction OR Middle Cerebral Artery Infarction OR Posterior Cerebral Artery Infarction OR Brain Ischemia* OR Ischemic Encephalopath* OR Cerebral Ischemia* OR Brain Hypoxia-Ischemia OR Cerebral Small Vessel Disease*	104268

	OR Cerebral Microangiopath* OR Intracranial Arterial Disease* OR Intracranial Arterial Disorder* OR Arterial Brain Disease* OR Brain Arterial Disease* OR Arterial Brain Disorder* OR Intracranial Hemorrhage* OR Posterior Fossa Hemorrhage* OR Brain Hemorrhage* OR Cerebral Hemorrhage OR Cerebral Intraventricular Hemorrhage OR Hypertensive Intracranial Hemorrhage OR Cranial Epidural Hematoma OR Subdural Hematoma OR Pituitary Apoplexy OR Subarachnoid Hemorrhage OR Brain Infarction* OR Brain Infarct* OR Anterior Circulation Brain Infarction OR Brain Venous Infarction* OR Anterior Cerebral Circulation Infarction OR Posterior Circulation Brain Infarction OR Lacunar Stroke* OR Lacunar Syndrome* OR Lacunar Infarction* OR Lacunar Infarct* OR Intracranial Vasospasm* OR Intracranial Angiospasm* OR Intracranial Vascular Spasm* OR Cerebral Vasospasm* OR Cerebrovascular Spasm* OR Cerebral Angiospasm* OR Cerebral Artery Spasm* OR Hemiplegia* OR Transient Hemiplegia* OR Monoplegia* OR Post-Ictal Hemiplegia* OR Crossed Hemiplegia* OR Flaccid Hemiplegia* OR Infantile Hemiplegia* OR Spastic Hemiplegia* OR Pareses OR Paraparesis OR Muscular Pares* OR Muscle Pares* OR Monopares* OR Lower Extremity Pares* OR Crural Pares* OR Upper Extremity Pares* OR Brachial Pares* OR Hemipares* OR Spastic Paraparesis OR Neurologic Gait Disorder* OR Neurologic Locomotion Disorder* OR Neurologic Ambulation Disorder* OR Neurologic Gait Dysfunction* OR Duck Gait OR Sensorimotor Gait Disorder* OR Athetotic Gait OR Broadened Gait OR Drop Foot Gait OR Festinating Gait OR Frontal Gait OR Hemiplegic Gait OR Hysterical Gait OR Reeling Gait OR Rigid Gait OR Scissors Gait OR Shuffling Gait* OR	

	Spastic Gait OR Stumbling Gait OR Unsteady Gait OR Widebased Gait OR Marche a Petit Pas OR Rapid Fatigue Gait OR Charcot Gait* OR Charcot* Gait OR Gait Apraxia OR Gait Ataxia):ti,ab,kw	
3	#1 OR #2	105247
4	[mh "Headache"]	2543
5	(Cranial Pain* OR Cephalgias OR Headaches OR Cephalodynias OR Head Pain* OR Cephalalgia* OR Cephalodynia OR Cephalalgia OR Ocular Headaches* OR Periorbital Headache* OR Hemicrania OR Unilateral Headache* OR Bilateral Headaches* OR Orthostatic Headache* OR Throbbing Headache* OR Vertex Headache* OR Sharp Headache* OR Generalized Headache* OR Retro-Ocular Headache* OR Retro Ocular Headache*):ti,ab,kw	8995
6	#4 OR #5	10941
7	(China OR Chinese OR Guangxi OR "inner Mongolia" OR Ningxia OR Tibet OR Xizang OR Xinjiang OR Heilongjiang OR Jilin OR Liaoning OR Hebei OR Shanxi OR Shandong OR Shaanxi OR Gansu OR Qinghai OR Sichuan OR Hubei OR Hunan OR Henan OR Anhui OR Zhejiang OR Jiangsu OR Guangdong OR Jiangxi OR Fujiang OR Guizhou OR Yunnan OR Hainan OR Han OR Hui OR Mongolia OR Zhuang OR Taiwan OR "Hong Kong" OR Macau):ti,ab,kw	50473
8	#3 AND #6 AND #7	29

Web of science: from inception to April 15, 2022

#	searches	results
1	TS=(Gait Disorders, Neurologic OR Paresis OR Vasospasm, Intracranial OR Stroke, Lacunar OR Basal Ganglia Cerebrovascular Disease OR stroke* OR Cerebrovascular	3,902,869

	Accident* OR Cerebrovascular Apoplexy OR Brain Vascular Accident* OR Cerebrovascular Stroke* OR Apoplexy OR Cerebral Stroke* OR Acute Stroke* OR Acute Cerebrovascular Accident* OR Brain Stem Infarctions OR Cerebral Infarction OR Hemorrhagic Stroke OR Ischemic Stroke OR Embolic Stroke OR Thrombotic Stroke OR Cardiovascular Diseases OR Vascular Diseases OR Carotid Artery Disease* OR Carotid Artery Disorder* OR Carotid Arterial Disease* OR Carotid Atheroscleros* OR Carotid Atherosclerotic Disease* OR Internal Carotid Artery Diseases OR Common Carotid Artery Diseases OR External Carotid Artery Diseases OR External Carotid Arterial Diseases OR Carotid Artery Thrombosis OR Cerebrovascular Disorder* OR Intracranial Vascular Disease* OR Intracranial Vascular Disorder* OR Cerebrovascular Disease* OR Brain Vascular Disorder* OR Cerebrovascular Occlusion* OR Cerebrovascular Insufficienc* OR Basal Ganglia Vascular Disease* OR Lenticulostriate Vasculopath* OR Lenticulostriate Vascular Disease* OR Vascular Lenticulostriate Diseases OR Basal Ganglia Hemorrhage OR Putaminal Hemorrhage OR Cerebral Infarct* OR Left Hemisphere Cerebral Infarction OR Subcortical Infarction* OR Posterior Choroidal Artery Infarction OR Anterior Choroidal Artery Infarction OR Right Hemisphere Cerebral Infarction OR CADASIL OR Multi-Infarct Dementia OR Anterior Cerebral Artery Infarction OR Middle Cerebral Artery Infarction OR Posterior Cerebral Artery Infarction OR Brain Ischemia* OR Ischemic Encephalopath* OR Cerebral Ischemia* OR Brain Hypoxia-Ischemia OR Cerebral Small Vessel Disease* OR Cerebral Microangiopath* OR Intracranial Arterial Disease* OR	

	Intracranial Arterial Disorder* OR Arterial Brain Disease* OR Brain Arterial Disease* OR Arterial Brain Disorder* OR Intracranial Hemorrhage* OR Posterior Fossa Hemorrhage* OR Brain Hemorrhage* OR Cerebral Hemorrhage OR Cerebral Intraventricular Hemorrhage OR Hypertensive Intracranial Hemorrhage OR Cranial Epidural Hematoma OR Subdural Hematoma OR Pituitary Apoplexy OR Subarachnoid Hemorrhage OR Brain Infarction* OR Brain Infarct* OR Anterior Circulation Brain Infarction OR Brain Venous Infarction* OR Anterior Cerebral Circulation Infarction OR Posterior Circulation Brain Infarction OR Lacunar Stroke* OR Lacunar Syndrome* OR Lacunar Infarction* OR Lacunar Infarct* OR Intracranial Vasospasm* OR Intracranial Angiospasm* OR Intracranial Vascular Spasm* OR Cerebral Vasospasm* OR Cerebrovascular Spasm* OR Cerebral Angiospasm* OR Cerebral Artery Spasm* OR Hemiplegia* OR Transient Hemiplegia* OR Monoplegia* OR Post-Ictal Hemiplegia* OR Crossed Hemiplegia* OR Flaccid Hemiplegia* OR Infantile Hemiplegia* OR Spastic Hemiplegia* OR Pareses OR Paraparesis OR Muscular Pares* OR Muscle Pares* OR Monopares* OR Lower Extremity Pares* OR Crural Pares* OR Upper Extremity Pares* OR Brachial Pares* OR Hemipares* OR Spastic Paraparesis OR Neurologic Gait Disorder* OR Neurologic Locomotion Disorder* OR Neurologic Ambulation Disorder* OR Neurologic Gait Dysfunction* OR Duck Gait OR Sensorimotor Gait Disorder* OR Athetotic Gait OR Broadened Gait OR Drop Foot Gait OR Festinating Gait OR Frontal Gait OR Hemiplegic Gait OR Hysterical Gait OR Reeling Gait OR Rigid Gait OR Scissors Gait OR Shuffling Gait* OR Spastic Gait OR	

	Stumbling Gait OR Unsteady Gait OR Widebased Gait OR Marche a Petit Pas OR Rapid Fatigue Gait OR Charcot Gait* OR Charcot* Gait OR Gait Apraxia OR Gait Ataxia)	
2	TS=(Headache* OR Head Pain* OR Cephalodynia* OR Cranial Pain* OR Cephalalgia* OR Cephalgia* OR Generalized Headache* OR Ocular Headache* OR Orthostatic Headache* OR Vertex Headache* OR Retro-Ocular Headache* OR Retro Ocular Headache* OR Sharp Headache* OR Throbbing Headache* OR Unilateral Headache* OR Hemicrania OR Bilateral Headache* OR Periorbital Headache* OR Slit Ventricle Syndrome)	189,009
3	TS=(China OR Chinese OR Guangxi OR "inner Mongolia" OR Ningxia OR Tibet OR Xizang OR Xinjiang OR Heilongjiang OR Jilin OR Liaoning OR Hebei OR Shanxi OR Shandong OR Shaanxi OR Gansu OR Qinghai OR Sichuan OR Hubei OR Hunan OR Henan OR Anhui OR Zhejiang OR Jiangsu OR Guangdong OR Jiangxi OR Fujiang OR Guizhou OR Yunnan OR Hainan OR Han OR Hui OR Mongolia OR Zhuang OR Taiwan OR "Hong Kong" OR Macau)	1,807,260
4	#1 AND #2 AND #3	1107

Embase: from inception to April 15, 2022

#	searches	results
1	'cerebrovascular accident'/exp OR 'basal ganglion hemorrhage'/exp OR 'brain hematoma'/exp OR 'brain hemorrhage'/exp OR 'brain infarction'/exp OR 'brain ischemia'/exp OR 'carotid artery disease'/exp OR 'cerebral artery disease'/exp OR 'cerebrovascular accident'/exp OR 'intracranial aneurysm'/exp OR 'occlusive cerebrovascular disease'/exp OR 'vertebrobasilar insufficiency'/exp OR 'brain embolism'/exp OR 'brain vasospasm'/exp OR 'artery dissection'/exp OR 'hemiplegia'/exp OR 'stroke patient'/exp OR	833,054

	'stroke unit'/exp OR 'paresis'/exp OR 'gait disorder'/exp	
2	'abnormal gait':ab,ti OR 'acute cerebrovascular lesion':ab,ti OR 'acute focal cerebral vasculopathy':ab,ti OR 'apoplex*':ab,ti OR 'arteria vertebrobasillaris insufficiency':ab,ti OR 'arterial dissection':ab,ti OR 'basal gangli* cerebrovascular disease':ab,ti OR 'basal ganglia haemorrhage':ab,ti OR 'basal ganglion haemorrhage':ab,ti OR 'brachial basilar insufficiency':ab,ti OR 'brain accident':ab,ti OR 'brain attack':ab,ti OR 'brain arterial insufficiency':ab,ti OR 'brain artery obstruction':ab,ti OR 'brain artery occlusion':ab,ti OR 'brain artery thrombosis':ab,ti OR 'brain bleeding':ab,ti OR 'brain blood flow disturbance':ab,ti OR 'brain circulation disorder':ab,ti OR 'brain embolus':ab,ti OR 'brain haematoma':ab,ti OR 'brain haemorrhage':ab,ti OR 'brain infarct*':ab,ti OR 'brain insult*':ab,ti OR 'brain isch*emic attack':ab,ti OR 'brain ischaemia':ab,ti OR 'brain microh*emorrhage':ab,ti OR 'brain phlebothrombosis':ab,ti OR 'brain thrombo*':ab,ti OR 'brain vascular accident':ab,ti OR 'brain vascular obstruction':ab,ti OR 'brain vasospasm':ab,ti OR 'carotid arterial disorders':ab,ti OR 'carotid arteriopathy':ab,ti OR 'carotid artery dis*':ab,ti OR 'carotid disease':ab,ti OR 'cerebral apoplexia':ab,ti OR 'cerebral artery occlusion':ab,ti OR 'cerebral artery thrombosis':ab,ti OR 'cerebral blood circulation disorder':ab,ti OR 'cerebral blood flow disorder':ab,ti OR 'cerebral circulat* disorder':ab,ti OR 'cerebral emboli*':ab,ti OR 'cerebral haematoma':ab,ti OR 'cerebral haemorrhage':ab,ti OR 'cerebral infarct*':ab,ti OR 'cerebral insult':ab,ti OR 'cerebral isch*emia':ab,ti OR 'cerebral microbleed':ab,ti OR 'cerebral thrombosis':ab,ti OR 'cerebral vascular accident':ab,ti OR 'cerebral vascular insufficiency':ab,ti OR 'cerebral vasospasm':ab,ti OR 'cerebro	584,353

	vascular accident':ab,ti OR 'cerebrovascular accident':ab,ti OR 'cerebrovascular arrest':ab,ti OR 'cerebrovascular circulation disorder':ab,ti OR 'cerebrovascular embolism':ab,ti OR 'cerebrovascular failure':ab,ti OR 'cerebrovascular infarction':ab,ti OR 'cerebrovascular injury':ab,ti OR 'cerebrovascular insu*':ab,ti OR 'cerebrovascular insu*':ab,ti OR 'cerebrovascular isch*emia':ab,ti OR 'cerebrovascular obliteration':ab,ti OR 'cerebrovascular obstruction':ab,ti OR 'cerebrovascular occlusion':ab,ti OR 'cerebrovascular thrombosis':ab,ti OR 'cerebrum embolism':ab,ti OR 'cerebrum vascular accident':ab,ti OR 'corpus callosum bleeding':ab,ti OR 'corpus callosum haemorrhage':ab,ti OR 'cortical infarction':ab,ti OR 'cva':ab,ti OR encephalorrhagia:ab,ti OR 'gait deviation*':ab,ti OR 'hematencephalon':ab,ti OR hemip*:ab,ti OR 'hemisphere infarct*':ab,ti OR 'interhemispheric hematoma':ab,ti OR 'intracerebral bleeding':ab,ti OR 'intracerebral haematoma':ab,ti OR 'intracerebral haemorrhage':ab,ti OR 'intracortical hemorrhage':ab,ti OR 'intracranial aneurysm':ab,ti OR 'intracranial artery thrombosis':ab,ti OR 'intracranial bleeding':ab,ti OR 'intracranial embolism':ab,ti OR 'intracranial hematoma':ab,ti OR 'intracranial hemorrhage':ab,ti OR 'intracranial thrombosis':ab,ti OR 'intracranial vasospasm':ab,ti OR 'isch*emic cerebral attack':ab,ti OR 'isch*emic seizure':ab,ti OR 'ische*mia cerebri':ab,ti OR 'isch*emic brain disease':ab,ti OR 'isch*emic encephalopathy':ab,ti OR 'musc* paresis':ab,ti OR 'neural isch*emia':ab,ti OR 'occlusive cerebrovascular disease':ab,ti OR 'paretic muscle':ab,ti OR 'partial paralysis':ab,ti OR stroke:ab,ti OR 'thrombosis cerebri':ab,ti OR 'vertebral basilar insufficiency':ab,ti OR 'vertebrobasilar artery insufficiency':ab,ti OR 'vertebrobasilar disease':ab,ti OR	

	'vertebrobasilar isch*':ab,ti OR 'vertebrobasilar syndrome':ab,ti	
3	#1 OR #2	965,395
4	'headache and facial pain'/exp	351,131
5	'headache disorders':ab,ti OR (headache:ab,ti AND 'facial pain':ab,ti)	5,392
6	#4 OR #5	351,350
7	'China':ab,ti OR 'Chinese':ab,ti OR 'Guangxi':ab,ti OR 'inner Mongolia':ab,ti OR 'Ningxia':ab,ti OR 'Tibet':ab,ti OR 'Xizang':ab,ti OR 'Xinjiang':ab,ti OR 'Heilongjiang':ab,ti OR 'Jilin':ab,ti OR 'Liaoning':ab,ti OR 'Hebei':ab,ti OR 'Shanxi':ab,ti OR 'Shandong':ab,ti OR 'Shaanxi':ab,ti OR 'Gansu':ab,ti OR 'Qinghai':ab,ti OR 'Sichuan':ab,ti OR 'Hubei':ab,ti OR 'Hunan':ab,ti OR 'Henan':ab,ti OR 'Anhui':ab,ti OR 'Zhejiang':ab,ti OR 'Jiangsu':ab,ti OR 'Guangdong':ab,ti OR 'Jiangxi':ab,ti OR 'Fujiang':ab,ti OR 'Guizhou':ab,ti OR 'Yunnan':ab,ti OR 'Hainan':ab,ti OR 'Han':ab,ti OR 'Hui':ab,ti OR 'Mongolia':ab,ti OR 'Zhuang':ab,ti OR 'Taiwan':ab,ti OR 'Hong Kong':ab,ti OR 'Macau':ab,ti	641,415
8	#3 AND #6 AND #7	726

PsycINFO (Ovid): from inception to April 15, 2022

#	searches	results
1	exp Ataxia/ or exp Basal Ganglia/ or exp Brain Disorders/ or exp Carotid Arteries/ or exp Cerebral Hemorrhage/ or exp Cerebral Ischemia/ or exp Cerebrovascular Accidents/ or exp Cerebrovascular Disorders/ or exp Gait/ or exp General Paresis/ or exp Hemiplegia/ or exp Movement Disorders/ or exp Paralysis/ or exp Cognitive Rehabilitation/ or exp Thromboses/ or exp Vasoconstriction/	269559
2	exp Migraine Headache/ or headache.mp. or exp Headache/ or exp	20201

	Muscle Contraction Headache/	
3	exp Cross Cultural Differences/ or exp Chinese Cultural Groups/ or exp Aging/ or exp Epidemiology/ or exp Age Differences/ or china.mp. or exp Rural Environments/	319294
4	1 and 2 and 3	242

the China Biomedical Literature Database (CBM): from inception to April 15, 2022

#	**searches**	**results**
1	"卒中"[不加权:扩展] OR "中风"[不加权:扩展] OR "垂体卒中"[不加权:扩展]<br>"Stroke" [Unweighted: Expanded] OR "Stroke" [Unweighted: Expanded] OR "Pituitary Stroke" [Unweighted: Expanded]	266549
2	"卒中"[常用字段:智能] OR "脑梗死"[常用字段:智能] OR "脑干梗死"[常用字段:智能] OR "脑血管意外"[常用字段:智能] OR "脑中风"[常用字段:智能] OR "腔隙性中风"[常用字段:智能] OR "脑静脉梗塞"[常用字段:智能] OR "脉络膜前动脉梗塞"[常用字段:智能] OR "皮层下梗塞"[常用字段:智能] OR "脉络膜后动脉梗阻"[常用字段:智能] OR "脑梗塞"[常用字段:智能] OR "多发性梗死性痴呆"[常用字段:智能] OR "大脑后动脉梗死"[常用字段:智能] OR "大脑中动脉梗死"[常用字段:智能] OR "大脑前动脉梗死"[常用字段:智能]<br>"Stroke"[Common Field:Intelligent] OR "Cerebral Infarction"[Common Field:Intelligent] OR "Brainstem Infarction"[Common Field:Intelligent] OR "Cerebral Vascular Accident"[Common Field:Intelligent] OR "Stroke"[Common Field: Smart] OR "Lacunar Stroke" [Common Field: Smart] OR "Cerebral Vein Infarction" [Common Field: Smart] OR "Prechoroidal	297215

	Infarction" [Common Field: Smart] OR "Subcortical Infarction" [Common field: smart] OR "retrochoroidal artery obstruction" [common field: smart] OR "cerebral infarction" [common field: smart] OR "multi-infarct dementia" [common field: smart] OR "posterior cerebral artery infarction" [Common Field: Smart] OR "Middle Cerebral Artery Infarction" [Common Field: Smart] OR "Anterior Cerebral Artery Infarction" [Common Field: Smart]	
3	(#2) OR (#1)	312737
4	"头痛"[不加权:扩展] OR "偏头痛"[不加权:扩展] OR "血管性头痛"[不加权:扩展] OR "紧张型头痛"[不加权:扩展] OR "头痛症"[不加权:扩展]<br>"headache" [unweighted: extended] OR "migraine" [unweighted: extended] OR "vascular headache" [unweighted: extended] OR "tension headache" [unweighted: extended] OR "headache disorder" [Unweighted: Extended]	35261
5	"裂隙脑室综合征"[常用字段:智能] OR "头部疼痛"[常用字段:智能] OR "偏头疼"[常用字段:智能] OR "偏头痛"[常用字段:智能] OR "先兆偏头痛"[常用字段:智能] OR "无先兆偏头痛"[常用字段:智能] OR "急性精神错乱性"[常用字段:智能] OR "偏头痛"[常用字段:智能] OR "偏头痛持续状态"[常用字段:智能] OR "头痛综合征"[常用字段:智能] OR "原发性头痛症"[常用字段:智能] OR "慢性头痛"[常用字段:智能] OR "继发性头痛症"[常用字段:智能]<br><br>"Slit Ventricular Syndrome" [Common Field: Smart] OR "Head Pain" [Common Field: Smart] OR "Migraine" [Common Field: Smart] OR "Migraine" [Common Field: Smart] OR "Migraine with Aura" headache" [common field: smart] OR "migraine without aura" [common field: smart] OR "acute delirium" [common field:	70221

	smart] OR "migraine" [common field: smart] OR "migraine persistent" Status" [Common Field: Smart] OR "Headache Syndrome" [Common Field: Smart] OR "Primary Headache Disorder" [Common Field: Smart] OR "Chronic Headache" [Common Field: Smart] OR "Secondary Headache" [Common Field: Smart] Headache "[Common Field:Intelligence]	
6	(#5) OR (#4)	70221
7	(#6) AND (#3)	3871

Note: English translation of above search strategies is shown below. Please note that Chinese literature retrieval should only be replicated using Chinese search terms.

the China National Knowledge Infrastructure (CNKI): from inception to April 15, 2022

#	searches	results
1	SU=(卒中+脑梗死+脑血管意外+脑血管中风+脑中风+腔隙性中风+脑静脉梗塞+脉络膜前动脉梗塞+皮层下梗塞+脉络膜后动脉梗阻+脑梗塞+多发性梗死性痴呆+大脑后动脉梗死+大脑中动脉梗死+大脑前动脉梗死)<br>SU = (stroke + cerebral infarction + cerebrovascular accident + cerebrovascular stroke + cerebral stroke + lacunar stroke + cerebral venous infarction + anterior choroidal artery infarction + subcortical infarction + posterior choroidal artery obstruction + cerebral infarction + multi-infarct dementia + Posterior cerebral artery infarction + middle cerebral artery infarction + anterior cerebral artery infarction)	350,837
2	SU=(头痛+偏头痛+血管性头痛+紧张型头痛+头痛症+裂隙脑室综合征+头部疼痛+偏头疼+偏头痛+先兆偏头痛+无先兆偏头痛+急性精神错乱性+偏头痛+偏头痛持续状态+头痛综合征+原	47,460

	发性头痛症+慢性头痛+继发性头痛症)<br>SU = (headache + migraine + vascular headache + tension-type headache + headache disorder + cleft ventricular syndrome + head pain + migraine + migraine + migraine with aura + migraine without aura + acute confusional + migraine + status migraine + headache syndrome + primary headache syndrome + chronic headache + secondary headache syndrome)	
3	#1 AND #2	1137

Note: English translation of above search strategies is shown below. Please note that Chinese literature retrieval should only be replicated using Chinese search terms.

Wangfang: from inception to April 15, 2022

#	searches	results
1	主题:(卒中 OR 脑梗死 OR 脑血管意外 OR 脑血管中风 OR 脑中风 OR 腔隙性中风 OR 脑静脉梗塞 OR 脉络膜前动脉梗塞 OR 皮层下梗塞 OR 脉络膜后动脉梗阻 OR 脑梗塞 OR 多发性梗死性痴呆 OR 大脑后动脉梗死 OR 大脑中动脉梗死 OR 大脑前动脉梗死)<br>Subject: (Stroke OR Cerebral Infarction OR Cerebral Vascular Accident OR Cerebral Vascular Stroke OR Cerebral Stroke OR Lacunar Stroke OR Cerebral Venous Infarction OR Anterior Choroidal Infarction OR Subcortical Infarction OR Retrochoroidal Artery Obstruction OR Cerebral Infarction OR Multi-Infarct Dementia OR posterior cerebral artery infarction OR middle cerebral artery infarction OR anterior cerebral artery infarction)	400523
2	主题:(头痛 OR 偏头痛 OR 血管性头痛 OR 紧张型头痛 OR 头痛症 OR 裂隙脑室综合征 OR 头部疼痛 OR 偏头疼 OR 偏头痛 OR 先兆偏头痛 OR 无先兆偏头痛 OR 急性精神错乱性 OR 偏头痛 OR 偏头痛持续状态 OR 头痛综合征 OR 原发性头	138477

	痛症 OR 慢性头痛 OR 继发性头痛症）<br>Subject: (Headache OR Migraine OR Vascular Headache OR Tension-Type Headache OR Headache Disorder OR Slit Ventricular Syndrome OR Head Pain OR Migraine OR Migraine OR Migraine With Aura OR Migraine Without Aura OR Acute Confusion OR Migraine OR status migraine OR headache syndrome OR primary headache OR chronic headache OR secondary headache)	
3	#1 AND #2	5745

Note: English translation of above search strategies is shown below. Please note that Chinese literature retrieval should only be replicated using Chinese search terms.

VIP: from inception to April 15, 2022

#	searches	results
1	M=(卒中 OR 脑梗死 OR 脑血管意外 OR 脑血管中风 OR 脑中风 OR 腔隙性中风 OR 脑静脉梗塞 OR 脉络膜前动脉梗塞 OR 皮层下梗塞 OR 脉络膜后动脉梗阻 OR 脑梗塞 OR 多发性梗死性痴呆 OR 大脑后动脉梗死 OR 大脑中动脉梗死 OR 大脑前动脉梗死)<br>M=(stroke OR cerebral infarction OR cerebrovascular accident OR cerebrovascular stroke OR cerebral stroke OR lacunar stroke OR cerebral venous infarction OR anterior choroidal artery infarction OR subcortical infarction OR posterior choroidal artery occlusion OR cerebral infarction OR multi-infarct dementia OR posterior cerebral artery infarction OR middle cerebral artery infarction OR anterior cerebral artery infarction)	219270
2	M=(头痛 OR 偏头痛 OR 血管性头痛 OR 紧张型头痛 OR 头痛症 OR 裂隙脑室综合征 OR 头部疼痛 OR 偏头疼 OR 偏头痛 OR 先兆偏头痛 OR 无先兆偏头痛 OR 急性精神错乱性 OR 偏头痛 OR 偏头痛持续状态 OR 头痛综合征 OR 原发性	32781

	头痛症 OR 慢性头痛 OR 继发性头痛症)<br>M=(headache OR migraine OR vascular headache OR tension-type headache OR headache disorder OR slit ventricle syndrome OR head pain OR migraine OR migraine OR migraine with aura OR migraine without aura OR acute delirium OR migraine OR status migraine OR headache syndrome OR primary headache OR chronic headache OR secondary headache)	
3	#1 AND #2	492

Note: English translation of above search strategies is shown below. Please note that Chinese literature retrieval should only be replicated using Chinese search terms.
