# Supplementary material for: Prevalence and risk factors of ischemic stroke-related headache in China: a systematic review and meta-analysis
Source: BMC Public Health. 2022 Aug 11;22:1533. doi: 10.1186/s12889-022-13917-z (PMC9367127; doi:10.1186/s12889-022-13917-z)
Supplement: Supplementary file 3 — Additional file 3. [file 12889_2022_13917_MOESM3_ESM.pdf]

Supplementary table 2. The modified Newcastle-Ottawa Scale.

(1) Representativeness of the sample:

- 1 point: Population contained a mixture of fields of study.
- 0 points: Population contained a single field of study.

(2) Sample size:

- 1 point: Sample size was greater than 200 participants.
- 0 points: Sample size was less than 200 participants.

(3) Non-respondents:

- 1 point: comparability between respondent and non-respondent characteristics was established, and the response rate was satisfactory (i.e., > 40%[**])
- 0 points: The response rate was unsatisfactory (i.e., < 40%), the comparability between respondents and non-respondents was unsatisfactory, or there was no description of the response rate or the characteristics of the responders and the non-responders.

(4) Ascertainment of headache:

- 1 point: a validated measurement tool was used.
- 0 points: no validated measurement tool was used, or the validation of the measurement tool was not described or referenced.

(5) Quality of descriptive statistics reporting:

- 1 point: Reported descriptive statistics to describe the population (i.e., age and gender distribution).
- 0 points: Descriptive statistics (i.e., age and gender distribution) were not reported, or were incomplete.

Legend: This scale, the scoring of which ranges from 0 to 5, assesses quality in several domains: sample representativeness and size, comparability between respondents and non-respondents, ascertainment of headache, and statistical quality.

* 40% is the mean response rate as reported in "Cook, C, Heath, F, & Thompson, RL. A Meta-Analysis of Response Rates in Web- or Internet-Based Surveys. Educational and Psychological Measurement. 2000; 60(6): 821–836".

(The individual components listed above are summed to generate a total bias risk score for each study, and the total scores ranged from 0 to 5 points. For the total score

grouping, studies were judged to be of low risk of bias (⩾3 points) or high risk of bias (<3 points).)
