# Supplementary material for: Prevalence and risk factors of ischemic stroke-related headache in China: a systematic review and meta-analysis
Source: BMC Public Health. 2022 Aug 11;22:1533. doi: 10.1186/s12889-022-13917-z (PMC9367127; doi:10.1186/s12889-022-13917-z)
Supplement: Supplementary file 4 — Additional file 4. [file 12889_2022_13917_MOESM4_ESM.pdf]

Supplementary table 3 Characteristics of the included studies.

No.	First Author, Year	Gender (female, N)	Age (Years)	Province; Area	Study Setting	Sample Size	Number of headaches	Diagnosis of Headache	Types of headaches	Quality
1	Wenyan Xie, 2021	288	PS: 59.0±8.43; NO PS: 57.0±10.8	Guangdong (S/Co)	U	630	164	NR	NR	3
2	Min Wang, 2021	22	Experimental group:46.9 ± 6.9; control group:45.2 ± 7.0)	Shandong (N/Co)	U	111	26	NR	Migraine	2
3	Yilin Pang 2021	36	1 month-15 years	Beijing (N/I)	U	109	10	NR	NR	8
4	Linqiang Hu, 2021	64	38.86±8.05	Hebei (N/Co)	U	137	14	Guidelines for the Prevention and Treatment of Migraine in China	Migraine	3
5	Mingzhu Gong, 2021	375	<45 years: 35.42±3.47; ≥45 years: 65.59±6.72	Jilin (N/I)	U	848	158	NR	NR	3
6	Yaxian Deng, 2021	69	9.0 (6.0-11.2)	Beijing (N/I)	U	172	36	NR	NR	6
7	Juanjuan Ran, 2021	127	59.7±11.6	Jiangsu (S/Co)	U	260	55	NR	NR	3
8	Deng, Y. 2021	69	1 month-18 years	Beijing (N/I)	Mixed	172	36	NR	NR	3
9	Hongjie Wang, 2020	50	64.3±4.3	Liaoning (N/Co)	U	120	65	NR	PIH	2

10	Yao Deng, 2020	68	Brain herniation: 65.8±11.8; no brain herniation: 69.4±11.3	Sichuan (S/I)	U	172	29	NR	NR	1
11	Dongping Chen, 2020	75	66(64,73)	Fujian (S/Co)	U	187	95	ICHD-3	Migraine, TTH	9
12	Yanmin zhang, 2019	28	56.34±3.07	Inner Mongolia (N/I)	U	68	30	NR	NR	8
13	Weiqing Zhang, 2019	39	66.48±12.33	Liaoning (N/Co)	U	140	8	NR	NR	1
14	Huixia Zhang, 2019	49	Older: 72.38±7.35; young and middle-aged: 42.19±7.13	Henan (N/I)	R	127	47	NR	NR	1
15	Li Wang, 2019	161	Youth: 39±5; middle-aged and older: 66±11	Beijing (N/I)	U	674	4	NR	Migraine	2
16	Feng Wang, 2019	12	29.8±3.4	Hebei (N/Co)	U	63	12	NR	NR	6
17	Xiaoyan Chen, 2019	159	63.1±13.9	Beijing (N/I)	U	515	36	ICHD-3β	POH, IIH, PIH	9
18	Zhenfei Cai, 2019	1774	≥65 years: 2217; <65 years: 1575	Zhejiang (N/Co)	U	3792	3127	NR	POH	9
19	Yuhan Wang, 2019	49	CAD: 51.56 ± 12.40; LAA: 55.45±8.06	Henan (N/I)	U	165	53	Visual analog scale	POH	3
20	Yingshuang Zhang, 2018	27	38.46±6.40	Beijing (N/I)	U	200	22	IHS	NR	4
21	Huiqin Liu,	24	PFO: 34±11; no	Henan (N/I)	U	104	11	NR	Migraine	2

	2018		PFO:35±12							
22	Xiaoqing Li, 2018	141	37±7	Beijing (N/I)	U	88	22	NR	Migraine	2
23	Huijun Zhao, 2017	77	SH: 65.75± 9.20; no SH: 66.87± 10.33	Shanxi (N/I)	U	183	56	NR	POH, IIH, PIH, TTH	2
24	Xiaolin Tan, 2017	24	54.39± 12.66	Sichuan (S/I)	U	138	14	NR	NR	2
25	Xiaojun Zheng, 2016	37	38.5±4.2	Shaanxi (N/I)	U	120	6	NR	Migraine	8
26	Yunhua Zang, 2016	44	65.12± 8.77	Shandong (N/Co)	U	100	8	NR	NR	4
27	Guangsheng Wang, 2016	125	58(40-83)	Jiangsu (S/Co)	R	433	115	Self-reported	Migraine	10
28	Hui Shen, 2016	35	88.4±3.5	Jiangsu (S/Co)	U	90	11	Self-reported	NR	10
29	Hao Lin, 2016	36	63±16	Inner Mongolia (N/I)	R	90	18	NR	POH	9
30	Yumei Chen, 2016	77	Young: 38.5±6.4; older: 73.2±10.2	Jilin (N/I)	U	260	114	NR	NR	3
31	Fei Zhuang, 2015	NR	83.8±5.5	Henan (N/I)	U	60	15	NR	POH	2
32	Yuanyuan Zhang, 2015	60	4.6±3.6	Henan (N/I)	U	92	9	NR	NR	2
33	Shiqi Yin, 2015	29	65±12	Heilongjiang (N/I)	R	62	15	NR	POH	7
34	Xuemei Wu, 2015	48	50-85	Anhui (S/I)	U	120	36	NR	NR	7

35	Jiafan Tang, 2015	317	NR	Jiangsu (Ce/Co)	U	755	514	NR	NR	1
36	Yafang Hu, 2015	30	60.5±11.2	Yunnan (S/I)	U	100	25	NR	Migraine	1
37	Lin Ren, 2014	79	Young and middle-aged: 34.23±3.21; older: 72.12±4.33	Henan (N/I)	U	209	64	NR	NR	2
38	Yali lv, 2014	26	Average age 61.1	Beijing (N/I)	U	67	12	NR	NR	7
39	Yuanyuan Zhang, 2013	142	67±8.1	Henan (N/I)	R	400	91	IHS (1988)	Migraine	1
40	Qiaorong Xu, 2013	43	40.56±5.20	Shaanxi (N/I)	Mixed	131	3	NR	Migraine	8
41	Qinhua Wu, 2013	239	Youth: 42.3±6.6; older: 71.0±10.5	Shanghai (S/Co)	R	536	9	NR	Migraine	3
42	Hui Liu, 2013	91	<60 years: 42.2±3.7; ≥60 years: 70.2±5.2	Hunan (S/I)	U	234	81	NR	NR	2
43	Huansong Guo, 2013	55	65-72	Shandong (N/Co)	R	100	52	NR	Migraine	7
44	Qing Zhai, 2013	27	39.27±4.04	Guangdong (S/Co)	U	112	8	NR	Migraine	2
45	Hao Cai, 2013	8	55-85	Hubei (S/I)	U	60	15	NR	NR	8
46	Junlin Bai, 2013	65	46±7.2	Henan (N/I)	R	156	49	NR	NR	1
47	Haijun Li, 2013	451	64.2±10.1	Zhejiang (S/Co)	U	968	166	IHS	Migraine, MA	4
48	Ping-Kun Chen, 2013	4503	61.0±14.3	Taiwan (S/Co)	Mixed	11523	848	ICHD-2	IIH	10

49	Yonghong Li, 2012	20	35.5±5.3	Guangdong (S/Co)	U	136	32	NR	POH	2
50	Yaxian Deng, 2012	21	6.99±5.60	Beijing (N/I)	U	81	10	NR	NR	8
51	Minquan Zhang, 2011	NR	NR	Jilin (N/I)	R	152	88	NR	NR	1
52	Junping Zhang, 2011	31	5l-78	Shanxi (N/I)	R	68	5	NR	NR	7
53	Peichun Song, 2011	54	39-76	Jilin (N/I)	U	199	30	NR	NR	6
54	Jinxia Li, 2011	30	45-80	Henan (N/I)	U	65	16	NR	NR	4
55	Hui Jiang, 2011	58	NR	Jiangsu (S/Co)	U	120	86	NR	NR	1
56	Jian Gao, 2009	37	49-78	Shaanxi (N/I)	U	70	15	NR	NR	4
57	Mingxiu Yang, 2008	64	36±4.65	Guangxi (S/Co)	U	186	22	NR	POH	6
58	Fengying Wu, 2008	21	15-45	Sichuan (S/I)	U	60	8	NR	NR	4
59	Guihong Tian, 2008	17	33-78	Jilin (N/I)	U	63	21	NR	NR	6
60	Ruihua Pan, 2008	117	38.46±7.02	Henan (N/I)	U	462	96	NR	POH	3
61	Ping Liu, 2008	23	3 months-15 years	Beijing (N/I)	U	65	12	NR	NR	7
62	Wei Hu, 2008	151	67.7±12.0	Jiangsu (S/Co)	U	371	51	NR	POH, IIH, PIH,	8

									TTH	
63	Hanbo Chen, 2008	258	PS: 59.0±8.4; no PS: 57.0±10.8	Guangdong (S/Co)	U	630	164	NR	NR	3
64	Yaoyi Yin, 2007	68	40-72	Hubei (S/I)	R	174	81	NR	NR	1
65	Shujuan Xiong, 2007	30	35-84	Hebei (N/Co)	R	66	18	NR	NR	6
66	Yiming Mo, 2007	76	28-82	Heilongjiang (N/I)	U	208	32	NR	NR	6
67	Shaoqing Li, 2007	47	21-45	Henan (N/I)	U	128	21	NR	NR	6
68	Chuanwei Cao, 2007	44	38-92	Guangxi (S/Co)	U	120	25	NR	NR	6
69	Xuejing Wang, 2006	24	45-86	Hunan (S/I)	U	80	11	NR	NR	4
70	Xiaobing Shi, 2006	76	63.9±7.9	Beijing (N/I)	U	277	54	NR	Migraine, POH, TTH	3
71	Shijie Zhang, 2005	27	16-45	Henan (N/I)	R	75	1	NR	NR	8
72	Lan Lin, 2005	54	36-87	Yunnan (S/I)	U	88	29	NR	NR	7
73	Jian Hu, 2005	61	63.5±4.9	Sichuan (S/I)	Mixed	154	25	NR	NR	8
74	Jianmei Zhang, 2004	72	22-86	Zhejiang (S/Co)	U	156	87	NR	NR	5

75	Zhou Zhang, 2003	53	15-45	Jiangsu (S/Co)	U	147	20	NR	NR	8
76	Yunlan Xu, 2003	58	42-83	Shandong (N/Co)	R	160	25	NR	NR	6
77	Xiaoxue Fu, 2003	49	32-84	Hainan (S/Co)	U	132	24	NR	NR	7
78	Jianmin Jiao, 2001	170	NR	Hebei (N/Co)	R	380	176	NR	NR	6
79	Taiyao Chen, 2001	30	NR	Hainan (S/Co)	R	88	45	NR	NR	6
80	Qifang Pan, 2000	21	62-86	Henan (N/I)	U	62	7	NR	NR	7
81	Xiuying Guo, 2000	41	Average age 61	Heilongjiang (N/I)	R	93	19	NR	Migraine	5
82	Zheng Wu, 1999	NR	NR	Fujian (S/Co)	U	116	27	NR	NR	2
83	Chaoping Niu, 1999	NR	NR	NR	NR	254	81	NR	POH, IIH, PIH	4
84	Yunyi Xu, 1998	NR	NR	Shanghai (S/Co)	U	300	49	NR	NR	7
85	Ziwen Wang, 1998	86	59.0±14.8	59.0±14.8	U	200	60	NR	NR	4
86	Dejun Mao, 1998	31	24-45	Shandong (N/Co)	U	125	11	NR	NR	7
87	Yanxiang Liu, 1998	60	60-86	Shanghai (Ce/Co)	U	132	30	NR	POH, IIH, PIH	7

88	Lianying Feng, 1998	116	42-89	Guizhou (S/I)	U	268	42	NR	NR	6
89	Duchu Wu, 1997	34	9-45	Shanghai (S/Co)	U	75	3	NR	Migraine	7
90	Wentao Li, 1997	33	23-80	Hebei (N/Co)	R	106	19	NR	NR	5
91	Qingyan Feng, 1997	42	38-81	Shandong (N/Co)	U	216	89	NR	NR	5
92	Suju Ding, 1997	NR	NR	Shanghai (S/Co)	U	312	24	Migraine diagnostic criteria developed by the Collaborative Group on Epidemiological Investigation of Neurological Disorders	Migraine, MA	4
93	Fengcun Ming, 1996	NR	NR	Shandong (N/Co)	U	295	25	Select Committee of the National institutes of Health	Migraine	4
94	Lijun Guo, 1995	60	32-84	Henan (N/I)	U	240	60	NR	NR	5
95	Yuanxiao Cui, 1995	27	26-76	Shandong (N/Co)	U	78	19	NR	POH	4
96	Xiaofei Chen, 1994	76	28-82	Beijing (N/I)	U	208	32	NR	NR	4

97	Zhanen Wang, 1993	9	64.94±5.76	NR	NR	79	10	NR	NR	6
98	Shoupeng Liu, 1985	NR	NR	Shanghai (S/Co)	U	137	34	NR	POH, PIH	6

N, north; S, south; I, inland; Co, coastal; U, urban; R, rural; PS, progressive stroke; NR, not reported; MA, migraine with aura; POH: pre onset headache; IIH, inter ictal headache; PIH, post ictal headache; TTH, tension type headache; PFO: patent foramen ovale; CAD: cervicocerebral artery dissection; LAA, large artery atherosclerosis; SH, stroke-associated headache; HIT-6, Headache Impact Test-6; ICHD-3, the International Classification of Headache Disorders, 3rd edition; ICHD-3β, the International Classification of Headache Disorder-3β; IHS, International Headache Society; ICHD-2, the International Classification of Headache Disorder, 2nd edition.

Reference:

1. Wenyan X, Yue Y, Zheng L. Early risk factors and nursing interventions for progressive stroke. *China Foreign Medical Treatment.* 2021;40:168-170.

2. Wang M, Zhao H, He X, Xie Q, Wei L. Clinical features and etiology of right-to-left shunt-associated cryptogenic stroke. *Advances in Clinical Medicine.* 2021;11:482-489.

3. Pang Y, Wu J, Zhang C*, et al.* Analysis of clinical features of arterial ischemic stroke in children. *Beijing Medical Journal.* 2021;43:1065-1069,1075.

4. Hu L, Xie D, Deng X, Zhao Z, Zheng K. Correlation between cryptogenic ischemic stroke and atrial septal distension tumor. *Stroke and Nervous Diseases.* 2021;28:410-414+430.

5. Gong M. Analysis of clinical characteristics and risk factors of cerebral infarction in young and middle aged people. *China Health Care & Nutrition.* 2021;31:23.

6. Deng Y, Zhang G, Wang L*, et al.* Clinical characteristics of ischemic stroke in children and current status of treatment. *Chinese Journal of Stroke.* 2021;16:816-821.

7. Ran J, Cui Y, Wang Y, Gu P. Relationship between fasting blood glucose and subsequent vascular events in Chinese patients with mild ischaemic stroke: a cohort study. *The Journal of international medical research*. 2021;49:3000605211019645.

8. Deng Y, Liu G, Zhang G*, et al.* Childhood strokes in China describing clinical characteristics, risk factors and performance indicators: a case-series study. *Stroke and vascular neurology*. 2021.

9. Wang H. Analysis of common causes and clinical characteristics of patients with massive cerebral infarction. *China Practical Medical.* 2020:34-36.

10. Deng Y, Zhang Y, zhou H*, et al.* Study of factors influencing brain herniation and prognosis in patients with massive cerebral infarction. *Practical Journal of Cardiac Cerebral Pneumal and Vascular Disease.* 2020;28:56-61.

11. Chen D, Hou S, Li H, Chen Y. Analysis of headache characteristics and risk factors in the post-infarction period. *Chinese Journal of Practical Nervous Diseases.*

2020;23:1307-1311.

12. Zhang Y. Clinical characteristics and clinical treatment effect of large cerebral infarction. *China Health Care & Nutrition.* 2019;29:121.

13. Zhang W, Cai J, Fan L, Wang J, Xin Y, Wang Y. Observation and analysis of the pattern of acute bilateral cerebellar infarction. *Chinese Journal of Geriatric Heart Brain and Vessel Diseases.* 2019;21:403-406.

14. Zhang H. Clinical characteristics and prognosis of light ischemic stroke in the elderly and young and middle-aged. *Clinical Research and Practice.* 2019;4:52-53.

15. Wang L, He X, Wang L, Liu G, Zhang Z. Risk factors and etiological typing analysis of young patients with cerebral infarction. *China Medicine*. 2019;14:1173-1176.

16. Wang F, Lu L, Lin N. Clinical characteristics of cerebral infarction in young people and analysis of the causes of misdiagnosis as simple partial-onset epilepsy. *Clinical Misdiagnosis & Mistherapy*. 2019;32:14-17.

17. Chen X, Su H, Zhang M*, et al.* Study on the correlation between ischemic stroke-related headache and infarct site. *Chinese Journal of Pain Medicine.* 2019;25:420-425.

18. Cai Z, Wu X, Cao D. Epidemiological characteristics of the occurrence of acute cerebral infarction in Taizhou region in 2017. *Practical Preventive Medicine.* 2019;26:1247-1250.

19. Wang Y, Cheng W, Lian Y. The Headache and Neck Pain in Ischemic Stroke Patients Caused by Cervicocerebral Artery Dissection. A Case-Control Study. *Journal of stroke and cerebrovascular diseases: the official journal of National Stroke Association*. 2019;28:557-561.

20. Zhang Y, Lu M, Chen L*, et al.* The relationship between headache and imaging features of cerebral small vessel disease in young stroke patients. *Chinese Journal of Stroke*. 2018;13:209-214.

21. Liu H, Liu L, Mei W, Wang C, Zhang J, Huang Y. Neuroimaging features of cryptogenic stroke with and without patent foramen ovale. *National Medical Journal of China.* 2018;98:2636-2640.

22. Li X, Yuan P, Ma W, Liu G. Clinical and imaging characteristics of young and middle-aged patients with cerebral infarction combined with patent foramen ovale. *National Medical Journal of China.* 2018;98:1507-1510.

23. Zhao H. Analysis of factors associated with the development of stroke-related headache in patients with lacunar cerebral infarction. *Chinese Journal of Integrative Medicine on Cardio/Cerebrovascular Disease.* 2017;15:100-102.

24. Tan X, Du D. Analysis on clinical characteristics and cerebral angiography results of artery dissection and large-artery atherosclerosis cerebral infarction. *Laboratory Medicine and Clinical.* 2017;14.

25. Zheng X, Liu J, Xiao F, Li H. Clinical analysis of risk factors and etiological subtypes in 120 cases of cerebral infarction in young people. *Shaanxi Medical Journal.* 2016;45:1526-1527.

26. Zang Y, Han T, Huang H, Wang Q, Zhao Y, Zhou X. The Comparison Study of Clinical Syndrome about Acute Ischemic Stroke and Chronic Cerebral Circulation Insufficiency. *Chinese Journal of Integrative Medicine on Cardio-/Cerebrovascuiar Disease.* 2016;14.

27. Wang G, Zhou Y, Chen X, Yang T, Ma X, Tong D. Epidemiological characteristics of symptomatic lacunar infarction in middle-aged and elderly people in Shuyang County, northern Su, China. *Chin J Clinicians (Electronic Edition).* 2016;10:1459-1464.

28. Shen H, Gong J. Clinical characteristics and their prognostic influences of ultra-high-aged patients with posterior circulation ischemic stroke. *Modern Diagnosis & Treatment.* 2016;27:3045-3046.

29. Lin H. Clinical characteristics and treatment analysis of massive cerebral infarction. *Yiayao Qianyan.* 2016;6:144-145.

30. Chen Y. Clinical analysis of 120 cases of stroke in young people. *China Practical Medicine.* 2016;11:108-109.

31. Zhuang F. An analysis of the characteristics of elderly patients with acute cerebral infarction. *Contemporary Medicine Forum.* 2015:215-216.

32. Zhang Y, Wang H. Risk factors and regression of arterial ischemic and

hemorrhagic strokes in children. *Journal of Clinical Pediatrics.* 2015:645-649.

33. Yin S, Yang G. Analysis of clinical characteristics and treatment of massive cerebral infarction. *China Continuing Medical Education.* 2015;7:53-54.

34. Wu X. Clinical analysis of 120 cases of lacunar cerebral infarction. *Journal of Bengbu Medical College.* 2015:1075-1076.

35. Tang J. Investigation of clinical characteristics and prognosis of ischemic cerebral infarction in young and middle-aged population. *Chinese Journal of Practical Nervous Diseases.* 2015:45-46.

36. Hu Y. Study on the correlation between migraine and lacunar cerebral infarction. *For All Health.* 2015:652-653.

37. Ren L, Li B, Li S. Comparison of clinical characteristics and risk factors of young and old patients with cerebral infarction. *Chinese Journal of Gerontology.* 2014:3451-3452.

38. Lv Y, Li J, Zhang Z. Analysis of clinical data of 67 patients with cerebellar infarction in a primary hospital. *Contemporary Medicine Series (second half of the month).* 2014:29-30,31.

39. Zhang Y, Li Y. Analysis of the correlation between migraine and cerebral infarction. *China Practical Medicine.* 2013:44-44.

40. Xu Q, Xu J, Li H, Zhao Z, Tang W. Analysis of risk factors and etiology of ischemic stroke in young people. *Chinese Journal of Modern Medicine.* 2013;23:95-97.

41. Wu Q, Geng Z, Zhang B*, et al.* Risk factors and prognosis of acute cerebral infarction in young patients. *Shanghai Medical Journal*. 2013;36.

42. Liu H, Xie M. Comparison and analysis of clinical characteristics of ischemic stroke in young and middle-aged and elderly patients. *Journal of Internal Intensive Medicine.* 2013;19:21-22,25.

43. Guo H. Analysis of clinical risk factors for asymptomatic cerebral infarction in elderly patients. *Guide of China Medicine*. 2013:347-347.

44. Zhai Q, Tong X. Analysis of risk factors in 112 cases of ischemic stroke in young people. Stroke and neurological diseases. *STROKE AND NERVOUS*

DISEASES. 2013;20:162-165.

45. Cai H. Study on the morbidity characteristics and clinical management of massive cerebral infarction. *Guide of China Medicine.* 2013:623-624.

46. Bai J. Study on the clinical characteristics of cerebral infarction. *HEALTHMUST-READMAGAZINE.* 2013;12:233-233.

47. Li H, Yu Y. Association between ischemic stroke and migraine in elderly Chinese: a case-control study. *BMC geriatrics*. 2013;13:126.

48. Chen P-K, Chiu P-Y, Tsai IJ*, et al.* Onset Headache Predicts Good Outcome in Patients With First-Ever Ischemic Stroke. *Stroke*. 2013;44:1852-+.

49. Li Y, Wang P, Zhao X. Clinical analysis of 68 cases of cerebral infarction in young people. *Chinese Journal of Postgraduates of Medicine.* 2012;35:83-85.

50. Deng Y, Gao B, Wang Y*, et al.* Analysis of risk factors and clinical characteristics of ischemic stroke in children. *Chinese Journal of Stroke.* 2012;7:448-452.

51. Zhang M. Clinical analysis of 76 cases of cerebral infarction in young people. *WORLD HEALTH DIGEST*. 2011;8:215-216.

52. Zhang J, Xu G. Treatment experience of 68 cases of large cerebral infarction in the acute phase. *China Health Industry.* 2011:86-87.

53. Song P. Clinical analysis of 30 cases of cerebral infarction with headache symptoms. *CHINA PRACTICAL MEDICAL*. 2011;6:93.

54. Li J. Clinical analysis of 65 cases of massive cerebral infarction. *Seeking medical advice (Academic Edition).* 2011;9:19-19.

55. Jiang H. Comparison of clinical characteristics and CT features of cerebral infarction in young and middle-aged people. *CHINA MEDICAL HERALD.* 2011;8:172-173.

56. Gao J, Shi Y. Clinical analysis of 70 cases of massive cerebral infarction. *Journal of Qiqihar Medical College.* 2009;30:2006.

57. Yang M, Chen H, Chen L. Clinical analysis of 186 cases of cerebral infarction in young people. *Chinese Journal of Modern Medicine.* 2008;18:219-221,224.

58. Wu F. Analysis of clinical characteristics and risk factors of 60 cases of cerebral

infarction in young people. *HAINAN MEDICAL JOURNAL*. 2008;19:72,76.
59. Tian G, Qin S. Clinical analysis of 63 patients with cerebellar infarction. *Contemporary Medicine (Academic Edition)*. 2008;14:9-11.
60. Pan R. Clinical features of 462 cases of ischemic stroke in young people. *Modern preventive medicine.* 2008;35:3877-3878.
61. Liu P, Zhang Y, Ma X*, et al.* Clinical characteristics and etiological analysis of ischemic stroke in children. *JOURNAL OF APPLIED CLINICAL PEDIATRICS.* 2008;23:1891-1893.
62. Hu W, Xiao G, Ge S, Yang J, Xu G, Liu X. Analysis of risk factors for lacunar cerebral infarction complicated by stroke-related headache. *Cinese Journal of Nervous and Mental Diseases.* 2008;34:262-265.
63. Chen H, Li C, Wu Z, Li L. Early risk factors and treatment ountermeasures for progressive stroke. *CHINESE JOURNAL OF POSTGRADUATES OF MEDICINE.* 2008;31:24-26.
64. Yin Y. Clinical analysis of 84 cases of massive cerebral infarction. *CHINA MODERN DOCTOR.* 2007;45:20-21.
65. Xiong S. Clinical analysis of 66 cases of patients with brainstem infarction. *SHANDONG MEDICAL JOURNAL*. 2007;47:28.
66. Mo Y. Exploring the clinical relationship between cerebral infarction and headache . Modern Health Care - Medical Innovation Research. *MODERN HEALTH(MEDICINE INNOVATION RESEARCH).* 2007;4:76-76.
67. Li S. Clinical analysis of 128 cases of ischemic stroke in young and middle-aged people. *HENAN MEDICAL RESEARCH.* 2007;16:219-221.
68. Cao C. Clinical analysis of 120 cases of asymptomatic cerebral infarction. *ACTA MEDICINAE SINICA.* 2007;20:85-86.
69. Wang X. Clinical analysis of 80 cases of asymptomatic cerebral infarction. *American Journal of Chinese Clinical Medicine.* 2006;8.
70. Shi X, Yu S, Lang S. Occipital lobe infarction and headache. *Chinese Journal of Pain Medicine.* 2006;12:270-273.
71. Zhang S. Clinical analysis of 75 cases of ischemic stroke in young people in

southern Henan area. *JOURNAL OF CHINESE COMMUNITY DOCTERS*. 2005:96.
72. Lin L, Zhang J, Ma S. Clinical Analysis of cerebral lobar infarction 88 cases. *Chinese journal of composite clinical medicine.* 2005;6.
73. Hu J, Bai X, Chen L. Clinical analysis of 154 cases of asymptomatic cerebral infarction. *SICHUAN MEDICAL JOURNAL*. 2005;26:1398-1399.
74. Zhang J, Gu S. Clinical analysis of occupational cerebral infarction. *JOURNAL OF ZHEJIANG COLLEGE OF TRADITIONAL CHINESE MEDICINE*. 2004;28:29-30.
75. Zhang Z, Sha R, Yuan H. Clinical analysis of 147 cases of ischemic stroke in young people. *JOURNAL OF CLINICAL NEUROLOGY.* 2003;16:301-302.
76. Xu Y, Yuan G, Ma Y. Clinical analysis of 160 cases of asymptomatic cerebral infarction. *CHINESE JOURNAL OF COAL INDUSTRY MEDICINE.* 2003;6:125-126.
77. Fu X, Wang X, Huang S. Clinical analysis of 132 cases of cerebral infarction. *CHINA TROPICAL MEDICINE.* 2003;3:344-344.
78. Jiao J, Song W, Li R. Clinical characteristics and CT analysis of cerebral infarction 380 cases. *HEBEI MEDICINE*. 2001;7:165-166.
79. Chen T. CT analysis of cerebral infarction 88 cases in the central mountainous region of Hainan Province. *HAINAN MEDICAL JOURNAL*. 2001;12:47.
80. Pan Q, Liu Q, Fan X, Jin J. Clinical characteristics and CT analysis of small amount of encapsulated cerebral hemorrhage in the elderly. *Journal of Clinical Medicine.* 2000;20.
81. Guo X, Ye L. Clinical analysis of headache characteristics of acute stroke 196 cases. *Heilongjiang Medical Journal.* 2000:47-48.
82. Wu Z, Lin L, Chen X. Analysis of risk factors for asymptomatic cerebral infarction. *CHINESE JOURNAL OF GERONTOLOGY*. 1999;19:114.
83. Niu C, Zhou P. Clinical characteristics and etiological analysis of headache in ischemic stroke. *MILITARY MEDICAL JOURNAL OF SOUTH CHINA*. 1999:22-23.
84. Xu Y, Xu F. Ischemic stroke with headache. *CHINESE JOURNAL OF CLINICAL NEUROSCIENCES*. 1998:39-41.

85. Wang Z, Guo A, Yan S. Clinical characteristics and the sodium valproate effect of headache in cerebral infarction. *ACTA NEUROPHARMA COLOGICA*. 1998:37.
86. Mao D, Tan Y, Wang Y. Analysis of cerebral infarction 125 cases in young people. *Journal of Practical Medicine.* 1998:291.
87. Liu Y, Lu W, Ye G. The clinical relationship between cerebral infarction and headache in the elderly. *HENAN MEDICAL INFORMATION*. 1998:22.
88. Feng L, Gui X. The relationship between headache and cerebral infarction. *Journalof Epileptology and Electroneurophysiology (China)*. 1998:62.
89. Wu D, Lv C, Cao P. Etiological analysis of cerebral infarction 75 cases in young people. *Chinese Journal of Nervous and Mental Diseases*. 1997:36-37.
90. Li W, Zhao F. Clinical relationship between cerebral infarction, headache and hypertension. Modern Journal of Integrative Medicine. *MODERN JORUNAL OF INTEGRATED TRADITIONAL CHINESE AND WESTERN MEDICINE*. 1997:1029.
91. Feng Q, Liu J, Zhao J. Clinical analysis of lacunar cerebral infarction 216 cases. *Journal of Jining Medical University.* 1997;20.
92. Ding S, Xu J, Zheng H. Clinical analysis of patients with migraine induced cerebral infarction. *Academic Journal of Naval Medical University*. 1997:97-98.
93. Ming F, Yang J, Han Z. Clinical analysis of migraine type stroke. *Stroke and Nervous Diseases.* 1996:42-43+45.
94. Guo L, Li Y, Zhu C. Analysis of 240 cases of acute ischemic stroke and headache. *Henan Medical Information*. 1995:30.
95. Cui Y, Yuan Q, Li Y. Early clinical features of brainstem infarction. *Journal of Brain and Nervous Diseases.* 1995;3.
96. Chen X, Pu C, Wang G. The clinical relationship between cerebral infarction and headache. *Chinese Journal of Practical Internal Medicine*. 1994:759.
97. Wang Z, Sun X. Clinical analysis of lacunar cerebral infarction 79 cases in the elderly. *Journal of Qingdao University School (Medical Sciences)*. 1993.
98. Liu S. Mechanism of headache in ischemic stroke (with clinical analysis of 34 cases). *Journal of Stroke and Nervous Diseases*. 1985;0.
